# Genetic and chemical disruption of amyloid precursor protein processing impairs zebrafish sleep maintenance

**DOI:** 10.1016/j.isci.2024.108870

**Published:** 2024-01-11

**Authors:** Güliz Gürel Özcan, Sumi Lim, Thomas Canning, Lavitasha Tirathdas, Joshua Donnelly, Tanushree Kundu, Jason Rihel

**Affiliations:** 1 Department of Cell and Developmental Biology, University College London, London, UK; 2 Department of Biostatistics and Health Informatics, Institute of Psychiatry, Psychology and Neuroscience, King’s College London, London, UK; 3 Social, Genetic & Developmental Psychiatry Centre, Institute of Psychiatry, Psychology & Neuroscience, King’s College London, London, UK

**Keywords:** Biological sciences, Molecular genetics, Molecular neuroscience

## Abstract

Amyloid precursor protein (APP) is a brain-rich, single pass transmembrane protein that is proteolytically processed into multiple products, including amyloid-beta (Aβ), a major driver of Alzheimer disease (AD). Although both overexpression of APP and exogenously delivered Aβ lead to changes in sleep, whether APP processing plays an endogenous role in regulating sleep is unknown. Here, we demonstrate that APP processing into Aβ40 and Aβ42 is conserved in zebrafish and then describe sleep/wake phenotypes in loss-of-function *appa* and *appb* mutants. Larvae with mutations in *appa* had reduced waking activity, whereas larvae that lacked *appb* had shortened sleep bout durations at night. Treatment with the γ-secretase inhibitor DAPT also shortened night sleep bouts, whereas the BACE-1 inhibitor lanabecestat lengthened sleep bouts. Intraventricular injection of P3 also shortened night sleep bouts, suggesting that the proper balance of Appb proteolytic processing is required for normal sleep maintenance in zebrafish.

## Highlights

• Zebrafish process APP into Aβ40 and Aβ42 at a conserved ratio (10–20:1)
• Larvae lacking *appb* exhibit shorter sleep bouts at night
• Blocking β- and γ-secretase cleavage alters night sleep in an *appb*-dependent manner
• Exogenous P3 shortens sleep bouts, suggesting a role in sleep maintenance

## Graphical abstract

**Figure:**
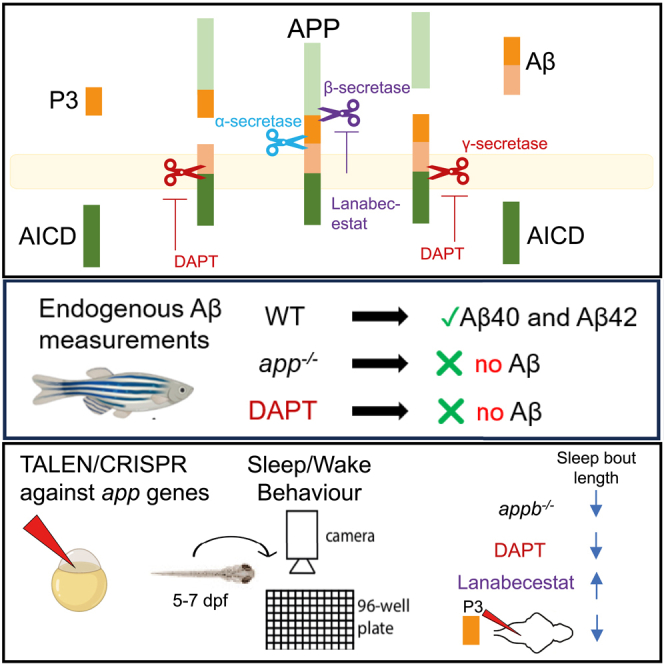

## Introduction

Sleep disturbances are prevalent in neuropsychiatric and neurodegenerative disorders such as Alzheimer disease (AD). In addition to cognitive impairment, individuals with AD experience altered sleep patterns, including reduced rapid eye movement (REM) and non-REM sleep, increased wakefulness, sleep fragmentation, and electroencephalogram (EEG) abnormalities.^1^ These sleep symptoms can manifest years before cognitive decline, and alterations in sleep can influence the deposition rates of amyloid-β (Aβ) plaques that are the hallmark of AD, contributing to disease risk.^2^^,^^3^^,^^4^ Consistent with this, some transgenic AD mouse models that overproduce Aβ display sleep disruptions prior to plaque formation, even without evident neuronal loss.^5^^,^^6^^,^^7^^,^^8^^,^^9^^,^^10^^,^^11^ Recently, human studies have found that harboring a high genetic risk for AD correlates with sleep changes, such as increased sleep rebound following sleep loss, even in young adults.^12^ These observations suggest that there may be underlying early biological processes important for sleep regulation that are governed by AD susceptibility genes and contribute to disease progression.

One of the risk genes for AD encodes the amyloid precursor protein (APP), a transmembrane protein that is proteolytically processed into multiple smaller fragments, including AD-associated fragments such as Aβ40 and Aβ42. Mutations in *App* are associated with both early- and late-onset AD^13^^,^^14^ including some alleles that are dominant and fully penetrant for early-onset AD.^15^ Duplications of the App gene, including those associated with trisomy 21 (Down syndrome), also confer increased AD risk.^16^ However, APP is associated with other phenotypes beyond AD and has been ascribed a variety of other physiological roles in cell adhesion, axon growth, synapse formation and function, and intracellular signal transduction.^17^^,^^18^^,^^19^ Additionally, APP is processed into fragments other than Aβ, including P3, sAPPα, sAPPβ, and the APP intracellular domain (AICD), but the endogenous roles of these peptides are poorly understood. Whether APP and its proteolytic fragments play a role in sleep regulation is unknown.

To explore the role of APP and its derivatives in sleep regulation, we introduced loss-of-function mutations into both zebrafish orthologs of the human APP gene, *appa* and *appb*, and evaluated their sleep-wake behaviors using a high-throughput behavioral assay.^20^ Zebrafish are a good model to address this question because they possess the complete App processing machinery, including α-secretase (ADAM10), β-secretase proteins (BACE-1 and 2), and the components necessary for γ-secretase complex assembly, including Presenilin-1 (PSEN-1), Presenilin-2 (PSEN-2), Presenilin enhancer 2 (PEN-2), Anterior pharynx-defective 1 (APH1), and Nicastrin (NCSTN).^21^^,^^22^^,^^23^^,^^24^^,^^25^ In addition, our previous work had revealed that depending on its structural configuration, Aβ can either increase or decrease sleep duration in larval fish by signaling through distinct receptors,^26^ suggesting that App-derived products may act as sleep signals in zebrafish. In *Drosophila*, elevated production of Aβ leads to sleep fragmentation, memory loss, and neuronal hyperexcitability,^27^^,^^28^^,^^29^ even though flies do not make Aβ natively. Thus, it is very important to disentangle the impact of toxic Aβ causing neuronal dysfunction, which can alter sleep even in species that do not produce Aβ, and the role of endogenous APP signaling events. By combining genetic analysis of *app* mutants with pharmacological interventions that block γ-secretase- and BACE-1-dependent cleavage of APP, we provide evidence that proteolytic processing of *appb* is required for maintaining sleep at night in zebrafish larvae.

## Results

### Characterizing zebrafish *appa* and *appb* and generating mutants

**Figure 1:**
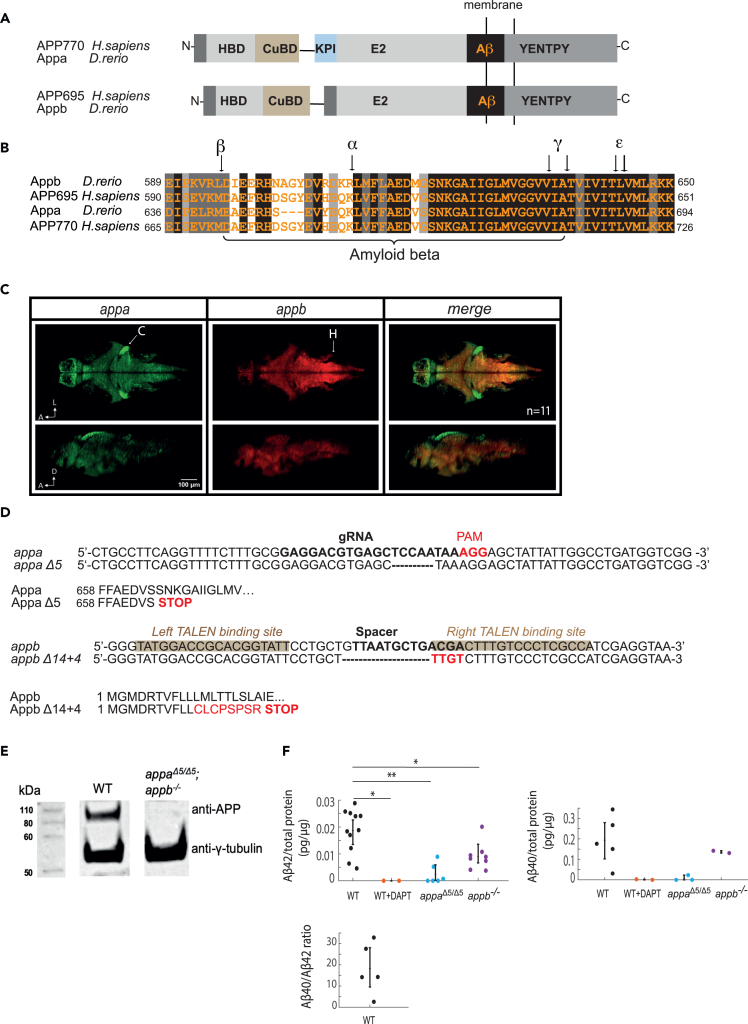
Zebrafish App protein organization, gene expression, and mutant generation (A) There are 2 *App* orthologs, *appa* and *appb*, in zebrafish. Appa contains the Kunitz-type protease inhibitor (KPI) domain and thus has a similar gene organization to the human APP770 isoform. Zebrafish Appb lacks the KPI domain similar to the brain-enriched human APP695 isoform. Both Appa and Appb have the functional App domains, including the heparin-binding domain (HBD), copper-binding domain (CuBD), extracellular E2 domain (E2), the conserved YENTPY motif, and amyloid-beta region (AB). (B) Alignment of Aβ regions of zebrafish Appa and Appb to human APP695 and APP770 shows high conservation within the Aβ region and the proteolytic cleavage sites (indicated with black arrows): α-secretase cleavage site (α), β-secretase cleavage site (β), γ-secretase cleavage sites (γ), and ε-cleavage sites (ε). Black, dark gray, and light gray boxes indicate strictly, highly, and moderately conserved amino acid residues, respectively. (C) As detected by multiplexed hybridization chain reaction (HCR), *appa* (green) and *appb* (red) are both expressed widely but in non-overlapping regions of the 5dpf larval brain, including the cerebellum and nuclei in the hindbrain (C, cerebellum; H, hindbrain). Shown is a representative image from a single brain taken at one z-plane (z140/420) (dorsal view, above) and through the midline of the same brain (lateral view, below) from an experiment with a total of n = 22 fish. A, anterior; D, dorsal; L, left. (D) CRISPR/Cas9 targeting of zebrafish *appa* resulted in a 5-bp deletion. The target guide RNA (gRNA) sequence is shown in bold, and the obligatory PAM sequence (AGG) is in red. The predicted translation of *appaΔ5* leads to a premature stop codon within the Aβ region (Appa amino acid 665). TALEN targeting of zebrafish *appb* resulted in a 14-bp deletion (dashes) and 4-bp insertion (red). The left and right sites targeted by the TALENs are highlighted, and the spacer sequence, where cleavage occurs, is bolded. The predicted translation of *appbΔ14+4* leads to a frameshift and a premature stop codon. (E) Western blot analysis of APP in brain homogenates from wild-type (WT) and *appa*^*Δ5/Δ5*^*; appb*^*−/−*^ double mutants. (F) Elisa detection of Aβ42 (left) and Aβ40 (right) levels in adult brain homogenates from WT controls, WT animals treated with the γ-secretase inhibitor DAPT for 24 h, *appa*^*Δ5/Δ5*^ and *appb*^*−/−*^ mutants quantified as picograms (pg) of Aβ per μg total protein extracted. Each dot is an independent biological replicate, and error bars represent the mean ± SEM. The bottom panel plots the ratio of Aβ40 to Aβ42 in WT zebrafish adult brain homogenates, n = 5. ∗p ≤ 0.05, ∗∗p ≤ 0.01, Dunnett’s test.

There are two *app* genes in zebrafish, *appa* and *appb*, that raise the question whether they have redundant functions. We first examined their relationship to the human APP isoforms, because gene duplications often take on isoform- or tissue-specific roles.^30^ Zebrafish Appa is more similar to the Kunitz-type protease inhibitor (KPI)-domain-containing APP751 and 770 isoforms, whereas the Appb protein lacks the KPI domain and is more similar to the APP695 isoform^31^^,^^32^ (Figures 1A and 1B). Both Appa and Appb have respectively an 80% and 71% conserved identity in the Aβ42 region compared with human APP, with conserved proteolytic cleavage sites for processing by α-, β-, and γ-secretases (Figure 1B). Both *appa and appb* genes are abundantly expressed in the zebrafish brain, although with non-overlapping expression patterns (Figure 1C). For example, while *appa* is expressed strongly in the cerebellum caudal lobe, olfactory bulb, and torus longitudinalis, *appb* is more strongly expressed in nuclei in the hindbrain (Figures 1C, S1, and S2). Examination of the brain expression pattern and levels of *appa* and *appb* during the day (4 h post lights on) and night (4 h post lights off) failed to detect any time-of-day differences, either globally or region specifically (Figures S2B and S3D). *appb* is also highly expressed in the very early stages of zebrafish development, indicating that it is maternally deposited (Figure S3A). Together, the gene expression patterns and structural homology differences of zebrafish Appa and Appb are consistent, with these proteins possibly having both isoform- and brain-tissue-specific functions.

We next generated zebrafish mutants with deletions in the *appa* and *appb* genes. To isolate mutations in *appa*, we used CRISPR/Cas9.^33^ The CRISPR design tool CHOPCHOP (http://chopchop.cbu.uib.no) was used to identify candidate gRNAs to target the conserved Αβ region (amino acid residues 25–35) of *appa*,^34^ which was then coinjected with Cas9 mRNA into zebrafish eggs at the one-cell stage. Injected animals (F0s) that harbored frameshift mutations were then identified by Illumina sequencing and outcrossed to wild-type animals to generate mutant families (see STAR Methods). One family was isolated that harbors a 5 base pair frameshift deletion (*appa*^*Δ5*^) that leads to an early stop codon within the Aβ domain. The *appa*^*Δ5*^ allele therefore lacks the conserved residues 26–42 of the Aβ and the entire intracellular C-terminus of Appa (Figures 1B and 1D).

To generate a loss-of-function mutation in the *appb* gene, two transcription activator-like effector nuclease (TALEN) arms targeting a conserved region within the first exon of the zebrafish *appb* gene were designed using the Zifit software (http://zifit.partners.org/ZiFiT/).^35^ These TALEN arms were each fused to one-half of a Fok1 heterodimer to generate mutagenic double-strand breaks within the first exon of *appb* (Figure 1D). F0 fish that had been coinjected at the one-cell stage with mRNA encoding the two TALEN arms were Illumina sequenced to identify a founder that contains an *appb* allele (*appb*^*Δ14+4*^) with a 14 base pair deletion and a 4 base pair insertion that generates a frameshift followed by an early stop codon. This founder was used to generate a stable heterozygous family (herein called *appb*^−/+^) for subsequent behavioral analysis (Figure 1D).

To confirm that these *appa* and *appb* alleles represent loss-of-function mutations, we performed western blot analysis on brain homogenates from adult double homozygous *appa* ^*Δ5/Δ5*^; *appb*^−/−^ mutants using an anti-APP antibody (22C11) that recognizes both zebrafish Appa and Appb. APP protein was detectable as a ∼100 kD band in wild type (WT) but not in *appa*^*Δ5/Δ5*^; *appb*^−/−^ double mutants, confirming that neither Appa nor Appb are made (Figures 1E and S3C). We also observed by RT-qPCR that mutant *appb* transcripts are only 25% of WT levels, consistent with non-sense-mediated decay that is often observed in transcripts harboring an early termination codon (Figure S3B).^36^

Although zebrafish have the machinery to process App into Aβ fragments, whether Aβ40/42 are actually generated in zebrafish has not been formally demonstrated. To measure endogenous Aβ levels, we used a highly sensitive electrochemiluminescence-based ELISA kit and detected in adult brains ∼0.02 pg Aβ42/μg total protein and ∼0.2 pg Aβ40/μg total protein, yielding an Aβ42:Aβ40 ratio of 1:15–1:20 (Figure 1F), similar to the range observed in various mammalian species.^37^ Aβ40 and Aβ42 were completely undetectable in brains following overnight exposure of WT adults to the γ-secretase inhibitor DAPT, confirming the efficacy of this drug to block Aβ40/42 production in zebrafish (Figure 1F). Moreover, levels of both Aβ40 and Aβ42 in either *appa*^*Δ5/Δ5*^ or *appb*^*−/−*^ mutants were significantly lower than in WT animals, suggesting that Aβ is made from both Appa and Appb (Figure 1F). The reduction of Aβ levels in *appa*^*Δ5/Δ5*^ mutants (−89% and −90% for Aβ40 and Aβ42, respectively) was stronger than in *appb*^*−/−*^ mutants, which may suggest that more Aβ is generated from Appa than from Appb; however, the Aβ40/42 epitopes detected by this kit are better matched in the Appa sequence than Appb, so the relative difference in detected Aβ levels may be due to slight differences in antibody affinity/detection between Appa and Appb. Nevertheless, these results are consistent with Aβ being produced from both Appa and Appb in zebrafish and demonstrate that both mutants have disruptions in Aβ production.

### *appa*^*Δ5/Δ5*^*and appb*^*Δ14+4*^*(appb*^*−/−*^*)* have distinct sleep-wake profiles

**Figure 2:**
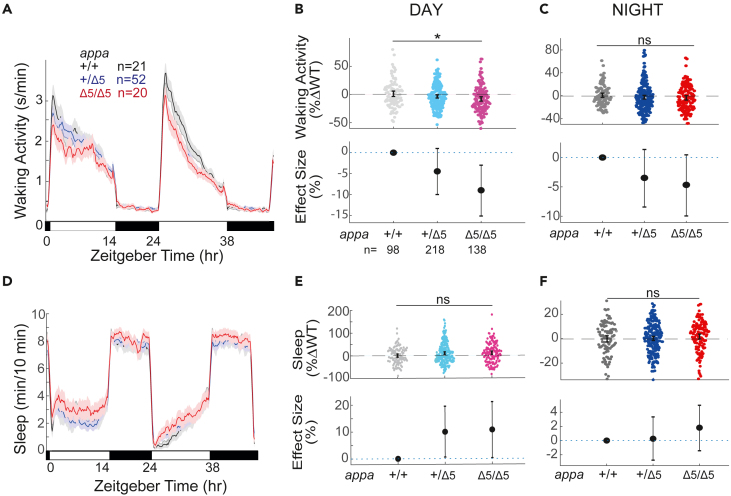
*appa*^*Δ5/Δ5*^ mutants have reduced day waking activity but no sleep phenotype Exemplar 48 h traces of average waking activity taken from a single experiment of *appa*^*Δ5/Δ5*^ mutants, heterozygous, and wild-type siblings (5–7dpf) on a 14 h:10 h light:dark cycle. Each line and shaded ribbon show the mean ± SEM. (B) Day waking activity and C) night waking activity for *appa*^*Δ5/Δ5*^ mutants and siblings from *appa*^*+/Δ5*^ incrosses, combined across N = 5 experiments. Each dot is a single larva, normalized to the mean of their experimentally matched WTs. At the bottom are plotted the effect sizes (±95% confidence interval [CI]) relative to WT. (D) Exemplar 48 h traces of average sleep for the same experiment shown in (A). (E) Day sleep and (F) night sleep of WT, heterozygous, and *appa*^*Δ5/Δ5*^ mutants normalized to their WT siblings, as in (B) and (C). ^ns^p > 0.05, ∗p ≤ 0.05, Kruskal-Wallis, Tukey’s post hoc test. n = number of larvae.

Zebrafish *appa*^*Δ5/Δ5*^ mutants do not have any obvious morphological abnormalities during development, have normal survival rates to adulthood, and are generally healthy and fertile. To examine whether *appa*^*Δ5/Δ5*^ mutant larvae have sleep or wake phenotypes, we used automated video monitoring to track larvae from in-crosses of *appa*^*Δ5/+*^ parents over several days on a 14h:10h light:dark cycle (Figure 2). Sleep in zebrafish is defined as a period of inactivity lasting longer than 1 min, as quiescent periods lasting at least this long are associated with an increased arousal threshold and other features of behavioral sleep, including circadian and homeostatic regulation.^38^ Zebrafish sleep is organized into bouts, with the sleep bout length describing the duration of consecutive, uninterrupted minutes of sleep. We also measured the vigor of their movements during the active bouts, quantified as the average waking activity. Sleep and waking activity are therefore not merely mirror-images and can be selectively and differentially modulated by drugs^20^ or mutation.^39^ Assessing these parameters across the day and night for *appa*^*Δ5/Δ5*^ mutants and their WT siblings uncovered subtle differences in mutant behavior. The *appa*^*Δ5/Δ5*^ mutant had a reduction of 9.0% (lower bound, −15.0%; upper bound, −3%, 95% confidence interval [CI]) in waking activity during the day compared with *appa*^*+/+*^ siblings (Figures 2A and 2B). At night, *appa*^*Δ5/Δ5*^ mutants also had slightly lower waking activity levels (−4.7%, [−10.0; −0.5, 95%CI]) (Figure 2C). In contrast, neither the total sleep (Figures 2E and 2F) nor the structure of sleep, such as the number and duration of sleep bouts (Figure S4), were statistically different across genotypes during either the night or the day.

Together, these data show Appa is not required for normal sleep states in zebrafish larvae, although it influences locomotor drive during the waking day.

**Figure 3:**
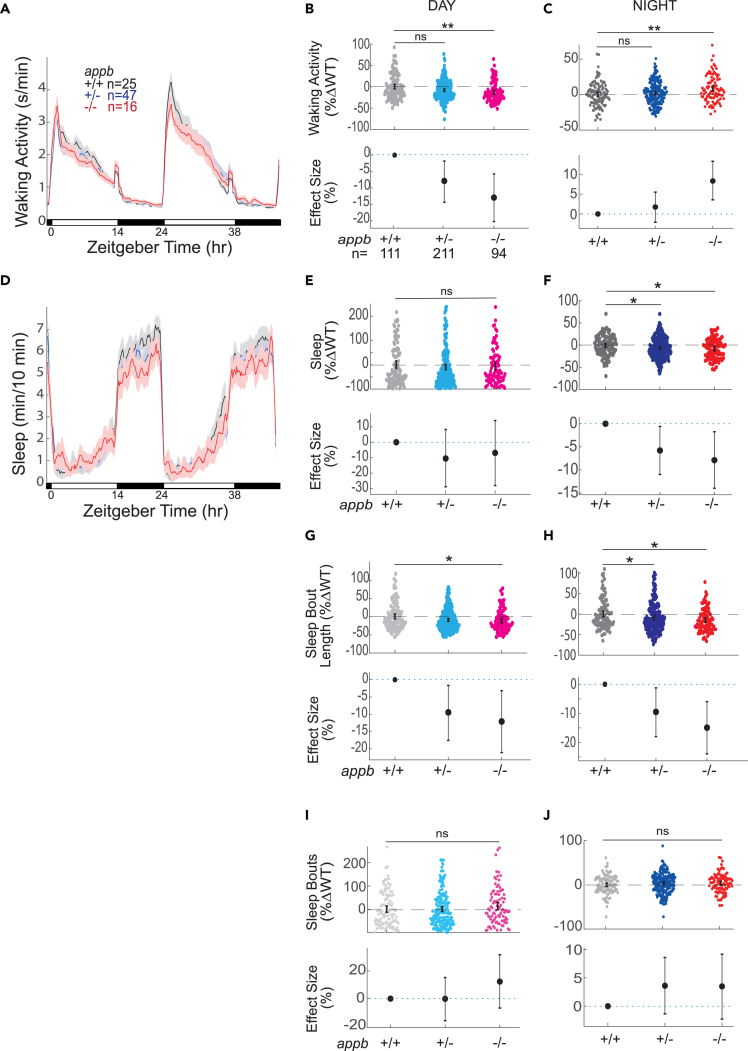
*appb*^*−/−*^ mutants have altered waking activity and sleep across the day-night cycle Exemplar 48 h traces of average waking activity taken from a single experiment of *appa*^*Δ5*^ mutants, heterozygous, and wild-type siblings (5-7dpf) on a 14h:10h light:dark cycle. Each line and shaded ribbon show the mean ± SEM. (B) Day waking activity and (C) night waking activity for *appb*^*−/−*^ mutants and siblings from *appb*^*+/−*^ incrosses, combined across N = 5 experiments. (D) Exemplar 48 h traces of average sleep for the same experiment shown in (A). (E) Day sleep, (F) night sleep, (G) day sleep length, (H) night sleep length, (I) day sleep bout number, and (J) night sleep bout number of WT*,* heterozygous, and *appb*^*−/−*^ mutants normalized to WT siblings as in (B) and (C). At top, each dot is an animal normalized to the mean of their experimentally matched WT. At bottom, shown are the effect size ±95%CI relative to WT. ^ns^p > 0.05, ∗p ≤ 0.05, ∗∗p ≤ 0.01, Kruskal-Wallis, Tukey’s post hoc test. n = number of larvae.

We also did not observe any obvious developmental delays or morphological abnormalities in *appb*^*−/−*^ mutant larvae or adults and therefore assessed whether Appb might play a non-redundant role in larval sleep regulation. Similar to *appa*^*Δ5/Δ5*^ mutants, *appb*^*−/−*^ larvae had a reduction in day-time waking activity of 13.0% [−20.2; −5.6, 95%CI] relative to *appb*^+/+^ siblings (Figures 3A and 3B). However, unlike *appa*^*Δ5/Δ5*^ animals, *appb*^*−/−*^ larvae had an increase in activity of 8.2% [3.3; 13.0, 95%CI] specifically at night (Figure 3C). We also observed that although *appb*^*−/−*^ larvae had unaffected sleep during the day (Figures 3D and 3E), they had a 7.9% (−14.0; −7.0, 95%CI) reduction in sleep at night (Figure 3F), which corresponds to ∼30 min less sleep per night. Thus, both Appa and Appb regulate daytime waking activity levels but have non-overlapping roles in regulating nighttime activity and sleep, with only *appb* mutants exhibiting sleep phenotypes.

To further investigate the nature of the decreased night sleep in *appb*^*−/−*^ mutants, we compared the sleep architecture of these mutants with their WT and heterozygous siblings. We specifically examined whether the change in total sleep was due to alterations in the number of sleep bouts (i.e., how often sleep is initiated) or in the average lengths of sleep bouts (i.e., once sleep is initiated, how long it is maintained). The average sleep bout length was shorter by 12.1% (−21.2; −3.2, 95%CI) in *appb*^*−/−*^ mutants during the day and by 14.9% (−23.9; −5.9, 95%CI) at night compared with their WT siblings (Figures 3G and 3H). The number of sleep bouts during either the day or night were not significantly different between *appb*^*−/−*^ mutants and WT animals (Figures 3I and 3J). These results show that the *appb*^*−/−*^ mutants initiate sleep normally but cannot sustain continuous sleep as long as WT, indicating a defect in sleep maintenance.

### γ- and β-secretase inhibitors modulate sleep maintenance in an appb-dependent manner

**Figure 4:**
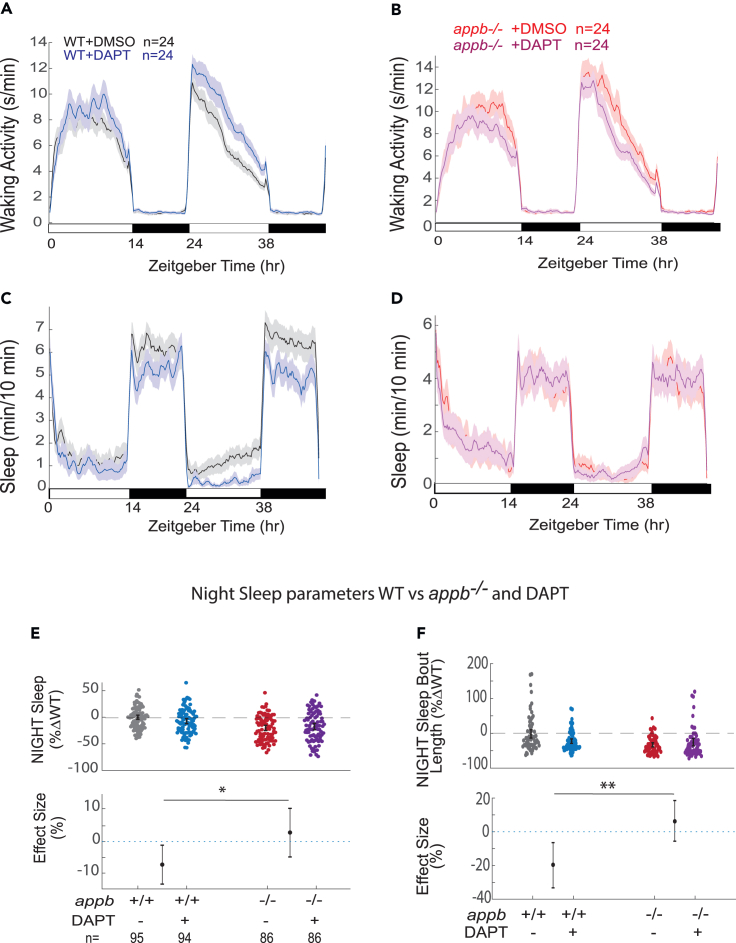
The γ-secretase inhibitor DAPT shortens sleep bout lengths at night in WT but not in *appb*^−/−^ mutants (A and B) Exemplar 48 h traces on a 14h:10h light:dark cycle of the average waking activity of WT (A) and *appb*^−/−^ mutants (B) continuously exposed to either 100 μM DAPT or DMSO vehicle control. (C and D) Exemplar 48 h traces of the average sleep from the same experiment shown in (A) and (B). (E) Night sleep and (F) night sleep bout length of WTs and *appb*^−/−^ mutants exposed to either 100 μM DAPT or DMSO vehicle. At top, each dot represents a single larva normalized to its experiment-matched WT (-DAPT) mean; error bars indicate ±SEM. At bottom, the within-genotype effect size and 95%CIs of DAPT treatment are plotted. n = the number of larvae. Data are pooled from N = 4 independent experiments, omitting the first day and night to account for any delay in drug action. ^ns^p > 0.05, ∗p ≤ 0.05, ∗∗p ≤ 0.01, two-way ANOVA, Tukey’s post hoc test. n = number of larvae.

Because App undergoes complex proteolytic processing, we decided to test whether drugs that inhibit App cleavage also modulate sleep in an Appb-dependent manner. We first tested whether the γ-secretase inhibitor DAPT, which effectively prevented Aβ production in zebrafish after 24 h (Figure 1F), alters sleep and waking activity in ether WT or *appb* homozygous mutants (Figures 4A–4D). Unlike either *appa* or *appb* mutants, which had lower waking activity during the day, DAPT significantly increased WT waking activity, with no effect at night (Figures 4A, S5A, and S5E). In contrast, DAPT significantly reduced daytime waking activity in *appb*^−/−^ larvae (Figures 4B and S5A). Together, these results indicate that γ-secretase-dependent cleavage products of Appb increase daytime waking, whereas other γ-secretase targets have a net effect of reducing wake activity.

DAPT also significantly reduced total nighttime sleep of WT larvae by 7.29% (−13.38; −1.06, 95%CI) and trended to lowered daytime sleep by 18.46% (−38.58; +0.87, %95CI) (Figures 4C–4E and S5B). This overall nighttime sleep reduction was due to a shortening in the average length of sleep bouts (−19.6% [-33.83; −6.55, %95CI], an effect size similar to *appb*^*−/−*^ alone), even though the number of sleep bouts was slightly increased by DAPT (Figures 4F and S5F). However, when DAPT was tested on *appb*^−/−^ mutants, there was no effect on total sleep or sleep bout lengths (Figures 4D–4F). This significant, non-additive effect (genotype × drug interaction, p < 0.05 for night sleep and p < 0.01 for sleep bout length at night, two-way ANOVA) cannot be explained by a flooring effect, as wake-promoting drugs can reduce zebrafish larval sleep much more than observed in *appb*^*−/−*^ mutants alone.^20^^,^^40^ Instead, this demonstrates that DAPT requires the presence of Appb to influence sleep length at night and further suggests that the short sleeping phenotype of *appb* mutants is due to the loss of Appb-derived γ-secretase cleavage products, such as Aβ, P3, or AICD.

**Figure 5:**
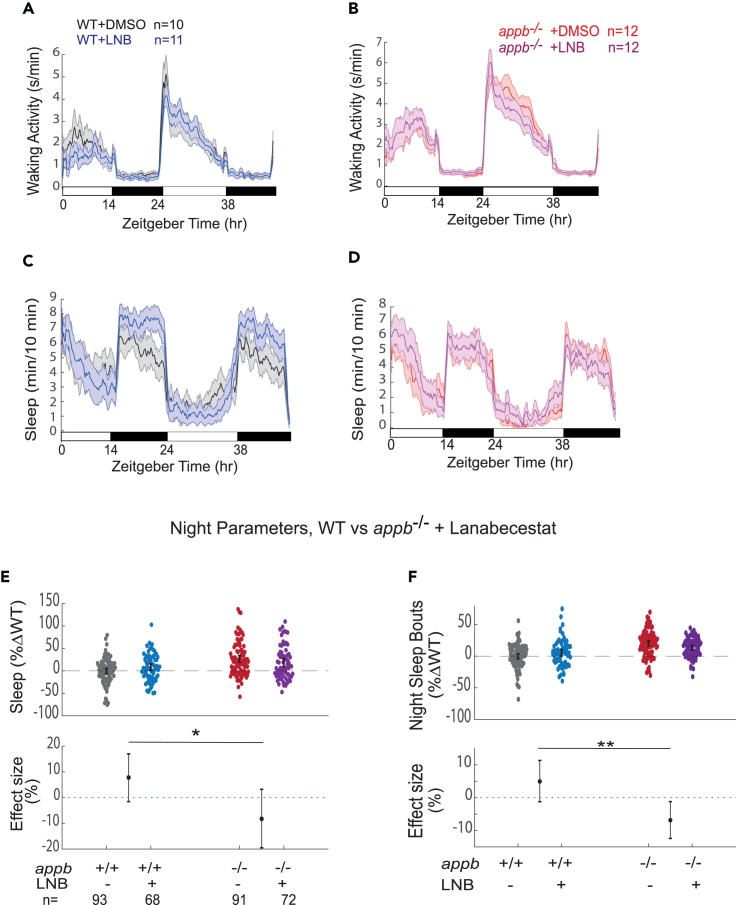
The β-secretase inhibitor lanabecestat increases sleep at night in WT but not in *appb*^−/−^ mutants (A and B) Exemplar 48 h traces on a 14h:10h light:dark cycle of the average waking activity of WT (A) and appb^−/−^ mutants (B) continuously exposed to either 0.3 μM lanabecestat or DMSO vehicle control. (C and D) Exemplar 48 h traces of the average sleep from the same experiment shown in A and B. (E) Night sleep and (F) night sleep bout length of WTs and *appb*^−/−^ mutants exposed to either 0.3 μM lanabecestat or DMSO vehicle. At top, each dot represents a single larva normalized to its experiment-matched WT (-lanabecestat) mean, and error bars indicate ±SEM. At bottom, the within-genotype effect size and 95%CIs of 0.3 μM lanabecestat treatment are plotted. n = the number of larvae. Data are pooled from N = 4 independent experiments, omitting the first day and night to account for any delay in drug action. ^ns^p > 0.05, ∗p ≤ 0.05, ∗∗p ≤ 0.01, two-way ANOVA, Tukey’s post hoc test. n = number of larvae.

To further dissect which Appb cleavage products modulate sleep and wakefulness, we next tested the effects of the BACE-1 inhibitor, lanabecestat, on WT and *appb*^−/−^ mutant behavior. Like γ-secretase inhibitors, blocking β-secretase will prevent Aβ production; however, other products blocked by γ-secretase inhibitors will remain unaffected, such as AICD, or even enhanced, such as P3, allowing us to test which Appb-derived products are responsible for shortening sleep (Table 1). Unlike DAPT but similar to *appa* and *appb* mutants, lanabecestat slightly reduced daytime waking activity of WT larvae (Figures 5A and S6A) and similar to *appa* mutants, reduced daytime waking activity at night (Figures S6A and S6E). However, when *appb* mutants were exposed to lanabecestat, there was no longer an effect on daytime waking levels, whereas nighttime waking activity was even slightly increased (Figures 5B and S6E). Thus, Appb must be present for β-secretase inhibition to exert an effect on daytime waking activity.

**Table 1:** Effects of blocking γ-secretase or β-secretase on proteolytic cleavage products on App and effects on night sleep

	APP	sAPPα	C83	P3	AICD	sAPPβ	C99	Aβ	Night sleep
*appb*^*−/−*^	**x**	**x**	**x**	**x**	**x**	**x**	**x**	**x**	
γ-secretase inhibition				**x**	**x**	**x**		**x**	
β-secretase inhibition					–	**x**	**x**	**x**	

, decrease; , increase; **x**, blocked production; –, no effect.^41^^,^^42^^,^^43^

Furthermore, unlike DAPT and *appb* mutants, lanabecestat increased sleep at night in WT larvae (+7.78, [−1.37; +17.06, %95CI,]) (Figures 5C–5E), due to an increase in both the number of sleep bouts (+4.95, [−6.34; +6.50, %95CI,]) and the average length of sleep bouts (+8.32, [−3.39; +20.45, %95CI]) (Figures 5E, 5F, and S6F). Similarly, lanabecestat was unable to increase total sleep, the number of sleep bouts, or the sleep bout lengths of *appb*^−/−^ mutants at night (Figures 5D–5F and S6F). The significant non-additive interaction between lanabecestat and *appb* genotype (genotype × drug interaction, p < 0.05 for night sleep and p < 0.01 for sleep bout number at night, two-way ANOVA) suggests that inhibition of β-secretase requires the presence of Appb to influence sleep at night.

### P3 brain injections reduce the length of sleep bouts at night

**Figure 6:**
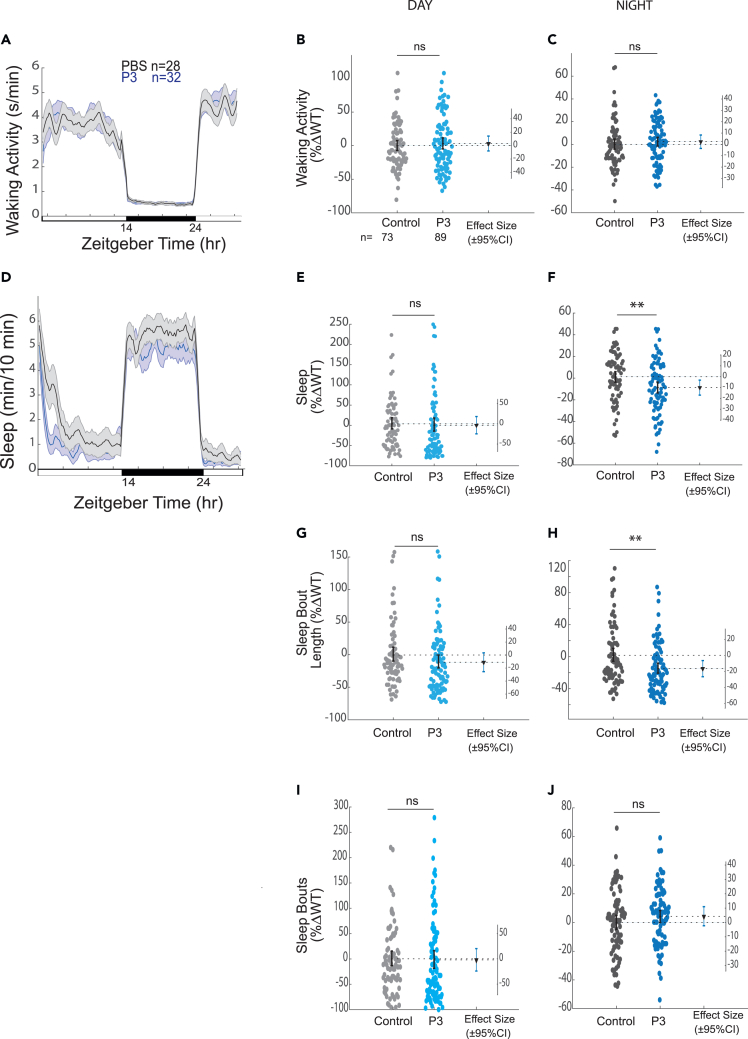
Intraventricular injection of P3 decreases sleep and shortens sleep bout lengths Exemplar 24 h traces of the average waking activity of WT larvae injected with either P3 or vehicle control on a 14h:10h light:dark cycle. Shown is a single exemplar experiment. (B and C) Average waking activity during the day (B) and night (C) across three independent experiments. Each dot represents a single larva normalized to the experiment-matched WT mean. The bars represent the mean. (D) Exemplar 24 h trace of the average sleep for the experiment shown in (A). (E) Day sleep, (F) night sleep, (G) day sleep bout length, (H) night sleep bout length, (I) day sleep bout number, and (J) night sleep bout number. n = the number of larvae. ^ns^p > 0.05, ∗p ≤ 0.05, ∗∗p ≤ 0.01, Kruskal-Wallis, Tukey’s post hoc test. n = number of larvae.

Blocking either β-secretase or γ-secretase resulted in Appb-dependent, but opposing, sleep phenotypes at night. Because β- and γ-secretase inhibition differentially alter the formation of App cleavage products, of which all are lost in *appb*^−/−^ mutants, we hypothesized that the nighttime sleep phenotypes might be explained by fragments that are blocked by γ-secretase inhibitors but enhanced or unchanged by β-secretase inhibition (Table 1). We therefore focused on P3, a partial Aβ fragment (Aβ_17-42_) that is boosted by BACE-1 inhibitors and absent when γ-secretase is blocked or *appb* is mutated. Injection of P3 into the brain ventricle of WT larvae had no effect on waking activity in either the day or the night (Figures 6A–6C) but caused a significant 10.03% (−15.05; −5.17, %95CI) decrease in night sleep relative to vehicle-injected controls (Figures 6D and 6F). As in other manipulations of App processing, this nighttime reduction in sleep was caused by shortened sleep bout lengths (−16.50% [−26.60; −6.30, %95 CI]) rather than a change in the number of sleep bouts (Figures 6G–6J). Although this demonstrates that App cleavage products such as P3 can have acute effects on sleep maintenance at night, because this effect is in the same direction as γ-secretase inhibition, which blocks the formation of P3, and in the opposite direction from BACE-1 inhibition, which enhances P3 production, alterations to P3 levels alone cannot explain the sleep phenotypes seen in *appb* mutants or drug manipulations that affect APP processing.

## Discussion

### Comparison to other App loss-of-function studies

We found that both *appa*^*Δ5/Δ5*^ and *appb*^*−/−*^ zebrafish had reduced locomotor activity during the day, but only *appb*^*−/−*^ animals had a reduction in sleep maintenance at night. Previous studies of mouse *App* knockouts have also observed reduced locomotor and exploratory activity, but many other phenotypes have been described, including reduced growth and brain weight, reduced grip strength, hypersensitivity to seizures, alterations in copper and lipid homeostasis, reactive gliosis, and impaired spatial learning.^44^^,^^45^^,^^46^^,^^47^^,^^48^^,^^49^^,^^50^^,^^51^^,^^52^^,^^53^ However, the lack of major morphological and developmental phenotypes in both *appa* and *appb* mutants stands in stark contrast to several reports that investigated zebrafish App function with morpholino knockdowns. For example, morpholino knockdown of zebrafish Appb has variously been reported to affect convergent-extension during gastrulation,^54^ axon outgrowth of spinal motor neurons,^55^^,^^56^ hindbrain neurogenesis,^57^ or cerebrovascular development.^58^ Our observations are more in line with other recent studies that do not find large developmental phenotypes for zebrafish *app* mutants.^59^^,^^60^^,^^61^ Because *appb*^−/−^ larvae from *appb*^−/−^ mothers also are morphologically normal (Figures 4 and 5), the difference in phenotype compared with morpholino studies cannot be explained by differential effects on maternally deposited transcripts of *appb* (Figure S3).

Although sleep has not been investigated in zebrafish *app* mutants before, two other studies have examined the role of *appb* on larval locomotor activity, coming to different conclusions than what we draw here. One *appb* morpholino study found that knockdown resulted in hyperlocomotion between 28 and 45 hpf,^55^ whereas we find both *appa*^*Δ5/Δ5*^ and *appb*^−/−^ larvae at older stages (4-7dpf) are less active than their WT siblings. These contrasting results are likely due to differences in locomotor behavior regulation at different stages of development, methodological differences (morpholino vs. knockout), or both. Another study of *appb*^−/−^ larvae tracked locomotor behavior for 60 min at a similar developmental age to our study (6 dpf) but found no differences in locomotion when comparing *appb* mutants with non-sibling WT animals.^59^ Given that we found *appa* and *appb* mutants are only 8%–10% less active than sibling-matched WT animals during the day, the short, 60-min observation window may not have been sufficient to capture this difference; alternatively, the time of day of observation might affect the ability to detect locomotor phenotypes (e.g., see Figures 2A and 3A). Indeed, we found *appb* mutants were significantly more, not less, active at night (Figure 3C).

Overall, our sleep and wake analysis of *appa* and *appb* mutants are broadly consistent with other rodent and zebrafish studies and expands the known phenotypes associated with App loss-of-function mutations. These results also demonstrate that Appa and Appb play at least partially non-redundant roles in the regulation of sleep and locomotor activity in larval zebrafish, which may explain why the zebrafish phenotypes of single mutants are somewhat milder than that reported for rodent App knockouts. Differences in the phenotypes between *appa* and *appb* mutants may reflect their differential expression patterns in the brain (Figures 1C, S1, and S2A), overall expression levels, or possibly different sensitivities or exposure to App processing enzymes, yielding different ratios of cleavage products from Appa versus Appb. For example, we found that Appa may be a larger source of Aβ in larvae, as *appa*^*Δ5/Δ5*^ mutants had lower detectable levels of Aβ compared with *appb*^−/−^ mutants (Figure 1F).

### Sleep maintenance and appb proteolytic cleavage

We observed no sleep phenotypes in *appa*^*Δ5/Δ5*^ mutants, but *appb*^*−/−*^ mutants had reduced nighttime sleep due to an inability to maintain longer sleep bout durations. Inhibition of γ-secretase also shortened the length of nighttime sleep bouts in an Appb-dependent manner, suggesting that γ-secretase-dependent cleavage products of Appb such as Aβ, P3, or AICD may act as a signal for maintaining nighttime sleep that is lost in *appb*^−/−^ mutants. Previous work has demonstrated that exogenously delivered Aβ does have both sleep-promoting and -inhibiting properties when injected into zebrafish larvae,^26^ engaging many areas of the brain including the sleep-promoting galanin-positive neurons of the preoptic area and hypothalamus.^62^ However, that work found that longer Aβ oligomers promoted sleep predominantly by increasing sleep initiation rather than altering sleep bout maintenance, whereas shorter forms of Aβ promoted wakefulness.^26^ Moreover, inhibition of BACE-1, which also prevents the formation of Aβ, instead led to increased sleep maintenance in an Appb-dependent manner. This suggests that loss of Appb-cleavage products other than Aβ is responsible for the short-sleeping phenotype of *appb*^−/−^ mutants.

One candidate App cleavage product that we tested was P3, as production of this peptide requires γ-secretase but is boosted by inhibition of BACE-1. Injection of P3 also reduced nighttime sleep by affecting sleep maintenance (Figure 6). Although this result underscores the potential of App-cleavage products to act as acute signals that regulate sleep, this contradicts the straightforward hypothesis that loss of P3 signaling is the cause of shortened nighttime sleep in *appb*^−/−^ mutants. However, because injection experiments do not recapitulate the precise timing or localization of P3 release, its local concentration, or (possibly) its structure, a role for P3, alone or in complex combinations with other App products like Aβ or AICD, in the observed *appb* mutant phenotypes cannot be completely ruled out. Future studies could examine whether mutations in components of γ-secretase, such as Presenilin-1 or Presenilin-2, or β-secretase, such as BACE-1, also have reduced sleep bout lengths at night. Zebrafish *bace1*^−/−^ mutants have been reported to have hypomyelination in the peripheral nervous system,^25^ but to date no sleep phenotypes have been described. As we performed here for *appb* and DAPT/lanabecestat, the examination of sleep phenotypes in *appb*^−/−^; *bace1*^−/−^ or *presenilin1/2*^−/−^ double mutants could be used to tease out which phenotypes are due to the specific cleavage of Appb.

Another possibility by which Appb could contribute to sleep regulation is raised by the recent observation that in zebrafish both Appa and Appb are colocalized to cilia and cells lining the ventricles at 30 h postfertilization.^60^ The *appa*^*−/−*^*; appb*^*−/−*^ double mutants were reported to have morphologically abnormal ependymal cilia and smaller brain ventricles.^60^ It would be interesting to see if the localization of App proteins to cilia and ventricles is important for sleep and locomotion, as the coordinated periodic beating of the cilia is involved in the generation of cerebrospinal fluid (CSF) flow within ventricle cavities,^63^ and CSF circulation is believed to facilitate transfer of signaling molecules and removal of metabolic waste products important for behavior.^64^^,^^65^

What emerges from these results is a complex picture of endogenous App-derived signals that can regulate sleep and wake in a bidirectional manner, with some signals boosting sleep and some inhibiting sleep. Changes in the relative composition of App-derived molecules, including both Aβ42 and P3 peptides (Aβ_17-40_ and Aβ_17-42_) over the progression of preclinical and clinical AD makes for even more complex scenarios. For example, P3 peptides (Aβ_17-40_ and Aβ_17-42_) are typically found in the diffuse plaques of individuals with AD,^66^ and cell culture experiments suggest that these peptides may be produced in even greater quantities than Aβ42 and Aβ40.^67^ Additionally, microglia in AD patients harbor various N-terminally truncated Aβ species,^68^ and the presence of P3 peptides in CSF shows a positive correlation with cognitive decline in AD patients,^69^ indicating a potential role of these peptides in AD pathogenesis. Our results suggest that in addition to Aβ,^26^^,^^70^ P3 might also interfere with sleep and wakefulness in AD and should be further investigated in rodent AD models.

Indicators of inadequate sleep quality, such as reduced sleep efficiency, extended time to fall asleep, heightened wakefulness during the night, and greater instances of daytime napping, have consistently been linked to compromised cognitive function.^71^^,^^72^^,^^73^^,^^74^ Furthermore, generation and release of Αβ42 into the interstitial fluid (ISF) is controlled by synaptic activity^75^ and even one night of sleep disruption can increase Aβ42 levels.^76^ As sleep can directly alter Αβ levels, sleep history over one’s lifetime may be a significant contributor to AD risk and progression. Our results are consistent with the idea that alterations in App gene products may be a direct contributor to sleep phenotypes associated with preclinical and clinical AD. The specificity of the effect of Appb loss on sleep architecture also suggests that specific changes in sleep patterns that could serve as a useful AD biomarker may yet be discovered.

### Limitations of the study

We have interpreted the lack of sleep effect of the γ-secretase (or β-secretase) inhibitors in the *appb*^*−/−*^ mutant background to be due to the loss of Appb proteolytic cleavage; however, it is technically possible that loss of Appb affects expression or localization of other γ-secretase targets (such as Notch), and the lack of drug-induced sleep alterations is due to this indirect effect. Another limitation of our study is that the exogenous ventricle P3 injections might not recapitulate the actual localization or structure of P3, and further experiments would be needed to dissect the roles of the App fragments.

## STAR★Methods

### Key resources table

**Table:** 

REAGENT or RESOURCE	SOURCE	IDENTIFIER
**Antibodies**		

Mouse anti-APP antibody (22C11)	Sigma-Aldrich	Cat#MAB348-AF647
Mouse anti-γ-tubulin monoclonal	Sigma-Aldrich	Cat#T6557; RRID: AB_477584
Secondary antibody anti-mouse-HRP	Sigma-Aldrich	Cat#A8924; RRID: AB_258426
Anti-Dig-AP	Roche	Cat# 11093274910; RRID: AB_514497

**Bacterial and virus strains**		

XL-10 Gold E. coli cells	Agilent	Cat#200315

**Chemicals, peptides, and recombinant proteins**		

Herculase II Fusion DNA polymerase	Agilent	Cat#600675
T4 DNA polymerase	NEB	Cat#M0203S
XbaI	NEB	Cat#R0145S
BamHI-HF	NEB	Cat#R3136S
SalI-HF	NEB	Cat#R3138S
MS-222	Sigma-Aldrich	Cat#A5040
ExoSAP-IT	ThermoFisher	Cat# 75001.1.ML
Lanabecestat	Cambridge Bioscience	Cat#HY-100740-2mg
TRIzol reagent	Ambion	Cat#15596026
T7 RNA polymerase	Roche	Cat#1109327491
HCR Amplifiers (B1-Alexa Fluor 488, B3-Alexa Fluor 546, and B5-Alexa Fluor 647)	Molecular Instruments	N/A
DAPT	Cell Guidance Systems	Cat#SM15-10
Protease inhibitors cocktail III	Calbiochem	Cat#539134
2% low melting point agarose	Thermo Fischer	Cat#16520100
4X NuPAGE LDS Sample Buffer	Thermo Fisher	Cat#NP0008
PBS	Oxoid	Cat# BR0014G
HFIP-treated Beta-Amyloid 17-42 peptide (P3)	Cambridge Biosciences	Cat#SP-Ab-23_0.1
NBT/ BCIP	Roche	Cat#11681451001

**Critical commercial assays**		

SuperScript III First-Strand Synthesis System	Invitrogen	Cat#18080051
Qiaquick PCR clean-up column	Qiagen	Cat#28104
MEGAscript SP6 kit	Ambion	Cat#AM1330
Qubit™ protein BR assay kit	Thermo Fisher	Cat#A50669
NuPAGE NOVEX 4-12% gradient Bis– TRIS pre-cast gel	Thermo Fisher	Cat#NP0321PK2
SuperSignal West Dura Extended Duration Substrate	Thermo Fisher	Cat#34075
GoTaq qPCR master mix	Promega	Cat#A6001
Qubit (dsDNA High Sensitivity Assay)	Thermo Fisher	Cat#Q33230
AffinityScript	Agilent	Cat#600559
DIG labelling kit	Roche	Cat# 11175033910
Illumina Sequencing MiSeq Reagent Nano Kit v2	Illumina	Cat# MS-102-2002
RNeasy Plus Micro Kit	Qiagen	Cat# 74134
KASP 2x Master Mix	LGC	Cat#KBS-1016-002
Pierce Detergent Compatible Bradford assay kit	Thermo Fisher	Cat#23246
Mesoscale Discovery V-plex Plus Aβ42 (4G8)	Meso Scale Discovery platform	Cat# K150SLG-1
Mesoscale Discovery V-plex Plus Aβ peptide panel 1 (4G8)	Meso Scale Discovery platform	Cat# K15199G-1

**Experimental models: Organisms/strains**		

Zebrafish: *appa*Δ5 (u539)	This paper	N/A
Zebrafish: *appb*Δ14+4 (u537)	This paper	N/A

**Oligonucleotides**		

Constant oligomer 5′AAAAGCACCGACTCGGTGCCACTTTTTCAAGTT GATAACGGACTAGCCTTATT TTAACTTGCTATTTCT AGCTCTAAAAC-3′	Gagnon et al.,^78^	N/A
oSQT34 5′-GACGGTGGCTGTCAAATACCAAGATATG-3′	Reyon et al.,^80^	N/A
oSQT35 5′-TCTCCTCCAGTTCACTTTTGACTAGTTGGG-3′	Reyon et al.^80^	N/A
oSQT1 5′-AGTAACAGCGGTAGAGGCAG-3′	Reyon et al.,^80^	N/A
oSQT3 5′- ATTGGGCTACGATGGACTCC-3′	Reyon et al.,^80^	N/A
oJS2980 5′-TTAATTCAATATATTCATGAGGCAC-3	Reyon et al.,^80^	N/A
Appa_miSeq_forward primer 5′TCGTCGGCAGCGTCAGATGTGTATAAGAGACAGCCTGCA GGAATAAAGCTGAT CT-3′	This paper	N/A
Appa_MiSeq_reverse primer, 5′-GTCTCGTGGGCTCGGAGATG TGTATAAGAGACAG ATGGACGTGTACTGCTTCTTCC-3′	This paper	N/A
Appb_PCR_F: 5’ - TCGTCGGCAGCGTCAGATGTGTATAAGAGA CAGCAG- CTGACTTTCCCTGGAGCA-3′	This paper	N/A
Appb_PCR_R: 5′-GTCTCGTGGGCTCGGAGATGTGTATAAGAGAC AG-TGGAGGAGAACCAAGCTCCTTC-3′	This paper	N/A
KASP primers for appa^Δ5^ 5′CTTTCTCTTTGTCTCCTGCCTTCAGGTT TTCTTTGCGGAGGACGTGAGC[TCCAA/]TAAAGGAGCTATTATTG GCCTGATGGTCGGAGGCGTCGTCATAGCAACCATCA TCGTCAT CACGCTGGTGATGCTGAGGAAGAAGCAGTACACGTCCATCCAC CACGGCATCATCGAGGTGCGTGAGTTCACACCGTCTCCAC-3′	This paper	N/A
KASP primers for appb^Δ14+4^ 5′-AAAATCGCGACAGAAAAACCCTGAT CCGCTCAGGATATATATDCACCAGGACGTGCTGCGCTTGGGAACA CAGCCATGGGTATGGACCGCACGGTATTCCTGCT[GTTAATGCTGA CGA/TTGT]CTTTGTCCCTCGCCATCGAGGTAAGAATGATTGTGTAA TGGAGAAGGAGCTTGGTTCTCCTCCATACTTTAAAGGGCGGCCA-3′	This paper	N/A
appa_forward_primer: 5′-CGCGGGTAAAGAGTCTGAGAGC-3′	This paper	N/A
appa-T7_reverse primer: 5′-TAATACGACTCACTATAGGG CAGACA GTATTCCTCCGACTC-3′	This paper	N/A
APPb_F: 5′-GCTCCAGGAGATATAAACGAAC-3′	This paper	N/A
APPb_T7R: 5′-TAATACGACTCACTATAGGGGCCGAACCTTTGGAAT CTCGG-3′	This paper	N/A
qPCR_appb_F2 5′-CGTGGTCATCGCTACTGTCA -3′	This paper	N/A
qPCR_appb_R2 (5′-CTGCCGCATCCACCTCAATA-3′	This paper	N/A
eef1a1_qRT-PCR Forward: 5′-TGCTGTGCGTGACATGAGGCAG-3′	Reichert et al.,^62^	N/A
eef1a1_qRT-PCR Reverse: 5′-CCGCAACCTTTGGAACGGTGT-3′	Reichert et al.,^62^	N/A

**Recombinant DNA**		

pT3TS-nCas9n (Addgene, plasmid 46757)	Addgene	RRID:Addgene_46757

**Software and algorithms**		

MATLAB (R2019a)	The Mathworks	N/A
Zifit software (http://zifit.partners.org/ZiFiT/)	Sander,^35^	N/A
CHOPCHOP (http://chopchop.cbu.uib.no)	Montague,^34^	N/A
ImageJ	ImageJ	N/A
Qiagen REST programme (2009)(v2.0.13)	Qiagen	N/A
GraphPad Prism	Dotmatics	N/A
MSD Mesoscale Discovery Workbench Toolbox	Mesoscale Discovery	N/A
zbb atlas (http://zbbrowser.com)	Tabor,^85^	N/A

### Resource availability

#### Lead contact

Further information and requests for resources and reagents should be directed to and will be fulfilled by the lead contact, Jason Rihel (j.rihel@ucl.ac.uk).

#### Materials availability

Zebrafish lines generated in this study [*appa*Δ5 (u539), line number 3257 and *appb*Δ14+4 (u537), line number 2590] have been sperm frozen and deposited to UCL zebrafish facility and are available upon request.

#### Data and code availability

All data reported in this paper will be shared by the lead contact upon request. All original code is available in this paper’s methods section. Any additional information required to reanalyze the data reported in this paper is available from the lead contact upon request.

### Experimental model and study participant details

Zebrafish (*Danio rerio*) husbandry and experiments were conducted following standard UCL fish facility protocols. Ethical approval for zebrafish experiments was obtained from the Home Office UK under the Animal Scientific Procedures Act 1986 under project licenses 70/7612, PA8D4D0E5, and PP6325955 awarded to JR and a personal licence 70/24631 to GGO. All zebrafish larvae were studied between 4-7 dpf well before the onset of sexual maturation.

### Method details

#### Zebrafish strains and husbandry

Zebrafish were raised under standard conditions at 28°C in a 14hr:10hr light:dark cycle. All zebrafish experiments and husbandry followed standard protocols of the UCL Fish Facility. *AB*, *TL and ABxTL* wild-type strains, *appa*^*Δ5*^ *(u539)* and *appb*^*Δ14+4*^*(*u537) (also called *appb*^*-/-*^ in this manuscript) were used in this study.

##### Generation of *appa* mutant

The *appa* gene was targeted by CRISPR/Cas9 mutagenesis. CHOPCHOP (http://chopchop.cbu.uib.no) was used to identify an sgRNA to exon 18 of *appa (*ENSDARG00000104279).^34^ Cas9 mRNA was made from pT3TS-nCas9n (Addgene, plasmid 46757)^77^ using mMESSAGE mMACHINE transcription kit (Thermofisher Scientific). Constant oligomer (5′AAAAGCACCGACTCGGTGCCACTTTTTCAAGTTGATAACGGACTAGCCTTATTTTAACTTGCTATTTCT AGCTCTAAAAC-3′) and the *appa* gene-specific oligomer targeting the conserved 25-42 amino acid region of Aβ in *appa* (target sequence: 5′-GAGGACGTGAGCTCCAATAA- 3′) were annealed on a PCR machine (using the program 95°C, 5 min; 95°C ->85°C, -2°C/second; 85°C ->25°C, -0.1°C/second, 4°C) and filled in using T4 DNA polymerase (NEB) using manufacturers’ instructions at 12°C for 20 min.^78^ The template was cleaned up using a PCR clean-up column (Qiaquick) and the 120 bp product was verified on a 2% agarose gel. The sgRNA was transcribed from this DNA template using Ambion MEGAscript SP6 kit.^78^ 1 nl of a 1 μl of Cas9 mRNA (200 ng/μl) and 1 μl purified sgRNA (25 ng/μl) containing mixture were co-injected into one-cell stage embryos. Injected F0 embryos were raised to adulthood, fin-clipped and deep-sequenced by Illumina Sequencing (below).

##### Generation of *appb* null mutant

The *appb* gene (ENSDARG00000055543) was disrupted using TALEN mutagenesis.^79^ Two TALEN arms targeting a conserved region within the first exon of zebrafish *appb* gene were designed using the Zifit software (http://zifit.partners.org/ZiFiT/)(Figure 1C)^35^ with Left Talen binding site sequence: 5′-TATGGACCGCACGGTATT-3′, Right TALEN binding site sequence: 5′-CGACTTTGTCCCTCGCCA-3′ and Spacer sequence: 5′-TTAATGCTGACGA-3’.

TALENs were generated using the FLASH assembly method following the protocol of 80. Starting with a library consisting of 376 plasmids that encode one to four TAL effector repeats consisting of all possible combinations of the NI, NN, HD or NG repeat variable di-residues (RVDs), the four 130 bp α-unit DNA fragments were amplified from each α-unit plasmid using the Herculase II Fusion DNA polymerase (Agilent) and oJS2581 and oJS2582 primers.^80^ The resulting 5′ biotinylated PCR products were digested with BsaI-HF (NEB) to generate four base-pair overhangs. To generate the DNA fragments encoding the βγδε (extension fragment) and βγδ (termination fragment) repeats, each of these plasmids was digested with BbsI followed by serial restriction digests of XbaI, BamHI-HF and SalI-HF (New England Biolabs) to cleave the plasmid backbone. The four TALEN expression vectors encoding one of four possible RVDs were linearised with BsmBI (NEB). The biotinylated α unit fragments were ligated to the first βγδε fragments using Quick T4 DNA ligase and bound to Dynabeads MyOne C1 streptavidin-coated magnetic beads (Life Technologies). The bead bound α-βγδε fragments were digested with BsaI-HF (NEB) to prepare the 3′ end of the DNA fragments for the subsequent ligation step. Each extension and termination fragment was then ligated to assemble the complete DNA fragment encoding the TALE repeat array by repeated digestion and ligation steps, and a final digestion with BbsI (NEB) released the full length fragments. The purified DNA fragments were ligated into one of four BsmBI (NEB) digested TALEN expression vectors encoding one of four possible RVDs using Quick T4 DNA ligase. Ligation products were transformed into chemically competent XL-10 Gold *E. coli* cells and clones grown on LB Agar plates containing Ampicillin at 37°C overnight. Bacterial colonies of each TALEN arm were selected and screened by colony PCR using primers oSQT34 (5′-GACGGTGGCTGTCAAATACCAAGATATG-3′) and oSQT35 (5′-TCTCCTCCAGTTCACTTTTGACTAGTTGGG-3′). Clones showing a correct sized band were cultured in LB medium containing Ampicillin at 37°C overnight. Following plasmid mini-preparation the inserts were sequenced using primers oSQT1 (5′-AGTAACAGCGGTAGAGGCAG-3′), oSQT3 (5′-ATTGGGCTACGATGGACTCC-3′) and oJS2980 (5′-TTAATTCAATATATTCATGAGGCAC-3′. mRNA was synthesised using the mMESSAGE mMACHINE T7 and polyA tailing kit. 100 pg of each of the TALEN mRNAs are injected into the cytoplasm of one-cell stage embryos, which were raised to adulthood and sequenced (below).

#### Sequencing/genotyping pipeline

F0 embryos were raised to adulthood, fin-clipped and deep-sequenced by Illumina Sequencing (MiSeq Reagent Nano Kit v2 (300 Cycles) (MS-103–1001)) to identify founders. Fin-clipping was done by anesthetizing the fish by immersion in 0.02% MS-222 (Tricaine) at neutral pH (final concentration 168 μg/ml MS-222). DNA was extracted by HotSHOT^81^ by lysing a small piece of the fin in 50 μl of base solution (25 mM KOH, 0.2 mM EDTA in water), incubated at 95°C for 30 min, then cooled to room temperature before 50 μl of neutralisation solution (40 mM Tris-HCL in water) was added. For *appa*, a 214 base pair fragment surrounding the conserved 25-35^th^ amino acid region within *appa* was PCR amplified using gene-specific primers with miSeq adaptors (forward primer, 5′-TCGTCGGCAGCGTCAGATGTGTATAAGAGACAGCCTGCAGGAATAAAGCTGATCT-3’; reverse primer, 5′-GTCTCGTGGGCTCGGAGATGTGTATAAGAGACAG ATGGACGTGTACTGCTTCTTCC-3′). The PCR program was: 95°C – 5 min, 40 cycles of [95°C – 30 s, 60°C – 30 s, 72°C – 30 s], 72°C – 10 min. For *appb,* founders were identified by PCR amplification using the primers (forward: 5’ -TCGTCGGCAGCGTCAGATGTGTATAAGAGACAGCAG-CTGACTTTCCCTGGAGCA-3’; reverse: 5′-GTCTCGTGGGCTCGGAGATGTGTATAAGAGACAG-TGGAGGAGAACCAAGCTCCTTC-3′). PCR program was 95°C – 5 min, 40 cycles of [95°C – 30 s, 60°C – 30 s, 72°C – 30 s], 72°C – 10 min.

The PCR product’s concentration was quantified with Qubit (dsDNA High Sensitivity Assay), then excess primers and dNTPs were removed by ExoSAP-IT (ThermoFisher) following the manufacturer’s instructions. The samples were then sequenced by Illumina MiSeq to assess the presence of insertion/deletions. The mutant F0 fish containing a 5 base pair deletion in the Αβ region resulting in a stop codon on the 26^th^ residue of Αβ (human numbering) was chosen to generate a stable mutant line *appa*^*Δ5*^ *(u539)*. An *appb* mutant carrier containing a 14 bp deletion and a 4 bp insertion *appb*^*Δ14+4*^ *(*u537) that is predicted to generate a frameshift and early stop codon was selected to make stable mutant lines for further analysis. F0 fish with indels were then outcrossed to wild-types and 10 one day old F1 embryos from each pairing were screened by Sanger sequencing to assess the nature of the mutations that passed into the germline. To minimize potential off-target mutations, mutant fish were crossed to ABxTL and TL WT strains for 3 generations before performing any behaviour experiments.

#### DNA extraction

Zebrafish DNA was extracted by the HotSHOT method.^81^ 50 μl of 1× base solution (25 mM KOH, 0.2 mM EDTA in water) was added to finclips in individual wells. Plates were sealed and incubated at 95°C for 30 min, cooled to room temperature and neutralised by adding 50 μl of 1× neutralisation solution (40 mM Tris-HCL in water). Genomic DNA was then stored at 4°C.

#### KASP genotyping

For rapid genotyping of mutant zebrafish harbouring the *appa*^*Δ5*^ and *appb*^*Δ14+4*^ alleles, a mutant allele-specific forward primer, a wild-type allele-specific forward primer and a common reverse primer were used for KASP genotyping (LGC Genomics, KBS-1050-102, KASP-TF V4.0 2X Master Mix 96/384, Standard ROX (25mL)). The primer sequences were as follows:

appa^Δ5^

5′-CTTTCTCTTTGTCTCCTGCCTTCAGGTTTTCTTTGCGGAGGACGTGAGC[TCCAA/- ]TAAAGGAGCTATTATTGGCCTGATGGTCGGAGGCGTCGTCATAGCAACCATCA TCGTCATCACGCTGGTGATGCTGAGGAAGAAGCAGTACACGTCCATCCACCACGGCATCATCGAGGTGCGTGAGTTCACACCGTCTCCAC-3’

appb^Δ14+4^

5′-AAAATCGCGACAGAAAAACCCTGATCCGCTCAGGATATATATDCACCAGGACGTGCTGCGCTTGGGAACACAGCCATGGGTATG

GACCGCACGGTATTCCTGCT[GTTAATGCTGACGA/TTGT]CTTTGTCCCTCGCCATCGAGGTAAGAATGATTGTGTAATGGAGAA

GGAGCTTGGTTCTCCTCCATACTTTAAAGGGCGGCCA-3’where [x/-] indicates the indel difference in [WT/mutant]. 8 μl of KASP reaction is run (3.89 μl 2x KASP reaction mix (http://www.lgcgroup.com/products/kasp-genotypingchemistry/reagents/mastermix/#.VgFRWd9VhBc, 0.11 μl KBD Specific Assay (primers), 1μl H2O, 3 μl (1:10 diluted) DNA) using the protocol (94°C for 15 minutes (Activation), 94°C 20 seconds, followed by 10 cycles of touchdown PCR (annealing 61°C to 53°C, decreasing 0.8°C per cycle), 94°C 20 seconds, then 26 cycles of standard 2-step PCR at 53°C 60 seconds.

Each genotyping plate contained three wells of negative controls (KASP mastermix only with no DNA) and three wells of positive controls (DNA of a known genotype: WT, Het, Homozygous). A minimum of 24 samples were used for good clustering.

Fluorescence was read on a CFX96 Touch Real-Time PCR Detection System (Bio-Rad) and the allelic discrimination plot generated using Bio-Rad CFX Manager Software.

##### Behavioural experiments

Behavioural tracking of larval zebrafish was performed as described in^20^^,^^82^ with the following adjustments. Zebrafish larvae were raised on a 14hr:10hr light:dark cycle at 28.5°C and at were placed into individual wells of a square-well 96-well plate (Whatman) containing 650 μL of standard embryo water (0.3 g/L Instant Ocean, 1 mg/L methylene blue, pH 7.0) at 4-5 dpf. Locomotor activity was monitored using an automated video tracking system (Zebrabox, Viewpoint LifeSciences) in a temperature-regulated room (26.5°C) and exposed to a 14hr:10hr white light:dark schedule with constant infrared illumination (Viewpoint Life Sciences). Larval movement was recorded using the Videotrack quantization mode. The movement of each larva was measured, and duration of movement was recorded with an integration time of 60 sec. Data were processed using custom PERL and MATLAB (The Mathworks, R2019a) scripts, and statistical tests were performed using MATLAB (The Mathworks, R2019a).

Any one-minute period of inactivity was defined as one minute of sleep.^82^ Sleep bout length describes the duration of consecutive, uninterrupted minutes of sleep whereas sleep bout number is the number of such sleep events in a given time interval. Average waking activity represents activity only during active periods.

All mutant larval zebrafish experiments were performed on siblings from *appa*^+/*Δ5*^ or *appb*^+/*Δ14+4*^ heterozygous incrosses, except for drug experiments, which were simultaneously performed on larvae from WT and *appb*^*Δ14+4/Δ14+4*^ incrosses from different parents. DAPT (Cell Guidance Systems, SM15-10) was dissolved in DMSO to make a stock concentration of 10 mM and diluted further to a working concentration of 10 μM in water in 1:10 serial dilutions. 6 μl of the 10 μM DAPT stock or 6 μl of 0.1% DMSO control was added individually to the wells in the behaviour plate to make a 100 nM final concentration the second day at Zeitgeber time 0 (Lights ON) of the video-tracking in each experiment. Lanabecestat (Cambridge Bioscience, HY-100740-2mg) was dissolved in DMSO to make a to make a stock concentration of 10 mM and diluted further to a working concentration of 100 μM in water in 1:10 serial dilutions. 1.8 μl of the 100 μM Lanabecestat stock or 1.8 μl of 1% DMSO control was added individually to the wells in the behaviour plate to make a 300 nM final concentration. The drug was added on the second day at Zeitgeber time 0 (Lights ON), when the larvae are 6 dpf.

#### Zebrafish *in situ* hybridization (ISH)

RNA was extracted from 30 WT embryos (5dpf) by snap freezing in liquid nitrogen and TRIzol RNA extraction (Ambion 15596026). 1 μg of RNA was reverse transcribed with AffinityScript (Agilent, 600559) to make cDNA following the manufacturer’s protocol.

Templates for *in vitro* transcription for *appa* and *appb* were generated by PCR using a reverse primer that contains a T7 promoter sequence (*appa* forward primer: 5′-CGCGGGTAAAGAGTCTGAGAGC-3′, *appa*-T7_reverse primer: 5′-TAATACGACTCACTATAGGG CAGACA GTATTCCTCCGACTC-3′, APPb_F: 5′-GCTCCAGGAGATATAAACGAAC-3′, APPb_T7R: 5′- TAATACGACTCACTATAGGG GCCGAACCTTTGGAATCTCGG-3′ using the 5 dpf cDNA library. 5 dpf larvae and 1,2,4,8 cell stage embryos were fixed in 4% paraformaldehyde (PFA) (with 4% sucrose for 5 dpf larvae) overnight at 4°C, dehydrated in a graded Methanol/PBST series (25%, 50%, 75%, 100%), kept overnight at -20°C and transferred into Phosphate Buffered Saline (PBS) the next morning. For 5dpf larvae, the brains were dissected by removing skin, cartilage, and eyes. A dioxigenin (DIG)-11-UTP-labeled antisense riboprobe targeting the gene transcript of interest was synthesized using the DIG labelling kit (Roche) and T7 RNA polymerase.^83^ Probe hybridization was carried out in hybridization buffer (50% formamide (v/v), 5x SSC (750 mM NaCl, 75 mM sodium citrate), 9.2 mM citric acid, 0.5 mg/mL Torula RNA, 0.05 mg/mL heparin, 0.1% Tween-20) supplemented with 5% dextran sulfate overnight at 65°C. The larvae were then incubated overnight at 4°C with anti-Dig-AP (1:2000 in 5% normal goat serum, 11093274910, Roche) and washed before detecting alkaline phosphatase using NBP/BCIP(Roche) according to the manufacture’s guidelines.

#### *In situ* hybridisation chain reaction (HCR)

##### Probe design

*In situ* Hybridization chain reaction (HCR) probes were designed as described.^84^ HCR split initiator sequences B1, B3 and B5 were taken directly from Choi et al. (2018).^84^ To generate optimal probe sets, probe pairs were excluded if they fell below the pre-set melting temperature and % GC thresholds. Probe pairs with strong sequence similarity to off-target transcripts were also excluded. For both *appa* and *appb* transcripts we generated probe sets that contained 20 probe pairs. HCR multiplexing was used to stain both paralogs in the same brain. HCR probes sets were purchased as custom DNA oligos from Thermo Fisher Scientific (UK), and HCR Amplifiers (B1- Alexa Fluor 488, B3- Alexa Fluor 546, and B5- Alexa Fluor 647) and buffers were purchased from Molecular Instruments (Los Angeles, CA, USA).

**Table:** Probe info

Gene	Transcript ID	Initiator	# probe pairs per set
*appa*	ENSDART00000170483.2	B3	20
*appb*	ENSDART00000077901.5	B5	20

#### Reaction protocol

*In situ* hybridization chain reaction (HCR) was performed following the “HCR RNA-FISH protocol for whole-mount zebrafish embryos and larvae” from Choi et al., 2018.^84^ The protocol was modified by omitting all proteinase K and methanol permeabilization steps. HCR multiplexing was performed by pooling probe sets targeted to *appa, appb*, and *gad1b* (*gad1b* was necessary to register brains to the zbb atlas, http://zbbrowser.com). WT 6dpf larvae that had been raised in 10hr:14hr normal or reversed light: dark cycle were euthanised post 4 hours lights on (for day condition) or lights off (for night condition) and fixed in 4% paraformaldehyde with 4% (w/v%) sucrose overnight at 4°C. Larvae were removed from the fixative by washing three times for 5 min in PBS before being transferred to a SYLGARD coated dish, where the eyes and the skin that covers the brain were removed with a pair of sharp forceps. After dissection, a 20 min postfix in 4% paraformaldehyde was performed followed by 3 further washes in PBST (1× DPBS + 0.1% Tween 20) to remove any fixative. Sample preparation was completed by two brief PBST washes. For the probe hybridization stage, larvae were first incubated in hybridization buffer in a heat block at 37°C for 30 min. The *appa* and *appb* probe solution was prepared by adding 4 μl of each 1 μM probe set to 500 μl hybridisation buffer (final probe concentration of 4 pmol). Hybridization buffer was replaced by the probe solution, and larvae were incubated at 37°C overnight (12–16 hr).

The next day, excess probe were removed by washing larvae four times 15 min in probe wash buffer preheated to 37°C, followed by two 5 min washes in 5x SSCT (5× sodium chloride sodium citrate + 0.1% Tween 20) at room temperature. Samples were kept at room temperature for subsequent amplification steps. Larvae were transferred to room temperature amplification buffer for 30 min. 3 μM stocks of hairpin H1 and hairpin H2 were individually snap cooled by heating to 95°C for 90 seconds then left to cool to room temperature in a dark drawer for 30 min. A hairpin solution was prepared by adding 10 μL of hairpin H1 and 10 μl of hairpin H2 to 500ul amplification buffer (final hairpin concentration of 30 pmol). Finally, pre-amplification buffer was removed and the hairpin solution was added to the larvae that were incubated overnight (12–16 hr) in the dark at room temperature. After overnight incubation, excess hairpins were removed by washing in 5× SSCT for 2 x 5 minute, then 2 x 30 minutes and finally 1 x 5 minute at room temperature. Larvae were transferred to PBS and kept at 4°C protected from light for up to 3 days.

#### Imaging

Following the HCR staining protocol, larvae were imaged using a ZEISS Lightsheet Z.1, fitted with a ZEISS W Plan-APOCHROMAT 10x/0.5 detection objective and two ZEISS LSFM 5x/0.1 illumination objectives. Larvae were mounted in glass capillary tubes filled with 1% low-melting agarose and held vertically, anterior facing up in a ZEISS Lightsheet Z.1 sample holder. HCR Amplifiers tagged with Alexa Fluor 488 (*gad1b*), Alexa Fluor 546 (*appa*), and Alexa Fluor 647 (*appb*) were imaged using 488 nm, 561 nm and 638 nm lasers, respectively. Full brain stacks were acquired with a final image size of 1920 x 1920 pixels (889.56 um x 889.56 um) and a voxel size of 0.46 um x 0.46 um x 1.00 um.

#### Registration

To allow for comparison of *appa* and *appb* expression among fish, all brains were registered onto the zbb brain atlas (http://zbbrowser.com)^85^ using the ANTs toolbox version 2.1.0.^86^ Raw Zeiss CZI files were rescaled, converted to 8-bit grayscale and saved in the NRRD file format. The *gad1b* channel from each *gad1b*-*appa*-*appb* hyperstack was registered to a *gad1b*-HCR reference brain (ref_gad1b) (1 × 1 × 1 xyz μm/px) using the following ANTs script:

fileext= nrrd

float= F1_app (e.g.)

ref= ref_gad1b (e.g.)

addstr = zbb (e.g.)

interp=BSpline

rigidm=GC

affinem=GC

synm=CC c

synp=[0.05,6,0.5]

antsRegistration -d 3 --float 1 -o [${float}_01${addstr}_,${float}_01${addstr}_Warped.nii.gz] -n ${interp} -r [${ref},${float}_01.${fileext}, 0] -t Rigid[0.25] -m ${rigidm}[${ref},${float}_01.${fileext},1,32,Regular,0.25] -c [200x200x200x0,1e-8,10] -f 12x8x4x2 -s 4x3x2x1 -t Affine[0.25] -m ${affinem}[${ref},${float}_01.${fileext},1,32,Regular,0.25] -c [200x200x200x0,1e-8,10] -f 12x8x4x2 -s 4x3x2x1 -t SyN${synp} -m ${synm}[${ref},${float}_01.${fileext},1,2] -c [200x200x200x200,1e-7,10] -f 12x8x4x2 -s 4x3x2x1.

The transformation files were applied to the *appa* and *appb* channels using the following ANTs script:

antsApplyTransforms -d 3 -v 0 --float -n ${interp} -i ${float}_0${i}.${fileext} -r ${ref} -o ${float}_0${i}${addstr}_Warped.nii.gz -t ${float}_01${addstr}_1Warp.nii.gz -t ${float}_01${addstr}_0GenericAffine.mat.

#### Western blots

Protein was extracted from adult *appa* ^*Δ5/ Δ5*^; *appb*^-/-^ mutants or WT larvae. Fish were euthanized, brains were dissected out and immediately frozen in liquid nitrogen prior to use and stored at − 80°C. Samples were homogenized in an ice-cold lysis buffer (10 mM Tris–HCl pH 8.0, 2% sodium deoxycholate, 2% SDS, 1 mM EDTA, 0.5 M NaCl, 15% glycerol) supplemented with protease inhibitors cocktail (Calbiochem protease inhibitors cocktail III) using a syringe needle (BD microlance, Ireland; 27G ½” 0.4 x 13 mm) on ice. Samples were then incubated 20 min on ice, sonicated for 10 min at 70% amplitude with a pulse of 30s on and off and then centrifuged at 10,000×*g* at 4°C. Supernatants were collected and kept on ice and protein concentration measured with a Qubit™ protein BR assay kit (Thermo Fisher Scientific, Waltham, MA) and samples stored at − 80°C. Protein samples (40-50ug) were then diluted in a denaturing lysis buffer (1X NuPAGE LDS Sample Buffer (Thermo Fisher Scientific, Waltham, MA), 0.05 M DTT (Sigma-Aldrich, St. Louis, MO), lysis buffer completed with protease inhibitors) and then boiled for 5 min at 95°C. Proteins were then separated on a NuPAGE NOVEX 4-12% gradient Bis–TRIS pre-cast gel (Thermo Fisher Scientific, Waltham, MA) at 150V for 1 hour and transferred onto a 0.2 μm Amersham™ Portran™ nitrocellulose membrane at 400 mA for 50 minutes on ice. The membrane was incubated in a blocking solution (5% milk) for 2 hours at RT and then immunoblotted overnight at 4°C with the primary mouse anti-amyloid precursor protein A4 antibody (clone 22C11) (Sigma MAB348-AF647) (1:3000) and with a loading concentration control mouse anti-γ-tubulin monoclonal (1:10,000) (Sigma, St. Louis, MO). The membrane was then washed in TBS-Tween three times for 10 min at RT and incubated with the secondary antibody anti-mouse-HRP (1:5000) (Sigma Aldrich) for one hour at RT. The signal was developed using SuperSignal West Dura Extended Duration Substrate kit (Thermo Fisher Scientific, Waltham, MA) and imaged using ChemiDoc Imaging (Bio-Rad, Hercules, CA). Western blot images were processed using ImageJ (NIH, USA).

#### qPCR

Total RNA was isolated from 4-6 dpf zebrafish larvae using the RNeasy Plus Micro Kit (Qiagen). cDNA was synthesized using the SuperScript III First-Strand Synthesis System (Invitrogen). qPCR was performed using a CFX96 machine (Bio-Rad) and accompanying BioRad CFX Manager (v3.1) using GoTaq qPCR master mix (Promega, A6001) with the primers qPCR_appb_F2 (5′-CGTGGTCATCGCTACTGTCA) and qPCR_appb_R2 (5′-CTGCCGCATCCACCTCAATA) at 60°C resulting in a 98 bp product. *ef1α* was chosen as the reference gene as it has been validated in the zebrafish for qPCR normalisation.^87^ Reactions were performed up to a total volume of 10μl per reaction with primer concentrations of 10μM for *ef1a, (eef1a1*_qRT-PCR Forward: 5′-TGCTGTGCGTGACATGAGGCAG-3′ and *eef1a1_*qRT-PCR Reverse: 5′-CCGCAACCTTTGGAACGGTGT-3′*)* or 20μM for *appb* primers. Efficiency of *ef1α* and *appb* primers were established to be within acceptable efficiency thresholds through a serial dilution series (90-110% efficiency). Threshold cycle values (Cq) were obtained for each gene in each sample in technical replicates. Two replicate experiments were performed per gene with 3 technical replicates.

#### qPCR analysis

Analysis was undertaken using the ‘delta-delta Cq’ method to compare the relative gene expression of the target gene (*appb*) to the reference gene (*ef1α*).^88^ Further analysis used the Qiagen REST programme (2009)(v2.0.13). This software compares treated versus untreated samples using provided serial dilution data for efficiency calculations. REST then performs a pairwise fixed reallocation randomisation test (permutations = 2000) to determine p values between samples, as well as giving confidence intervals and standard error of the permutation analysis. Melt curve analysis was conducted through the BioRad CFX manager software. Boxplots were generated using GraphPad Prism (Dotmatics).

##### Endogenous Aβ measurements in adult zebrafish brains

For the DAPT treatment, WT adult fish were treated with a final concentration 25 μM of DAPT for 24 hours in a small tank. Adult zebrafish brains were dissected, weighed and were mechanically homogenized in 100 μL TBS (50mM Tris-HCL, pH 8.0) containing Calbiochem protease inhibitor cocktail set III (1:200). Whole brain homogenates were centrifuged at 16,000 g at 4°C for 30 min, the supernatant was aliquoted and stored at -80°C. Total protein concentration of the samples was determined using a Pierce Detergent Compatible Bradford assay kit according the manufacturer’s instructions (Thermofisher, 23246). Aβ40 and Aβ42 measurements of the samples were done according to the manufacturers protocols using Mesoscale Discovery V-plex Plus Aβ42 (4G8) or V-plex Plus Aβ peptide panel 1 (4G8) kits on the Meso Scale Discovery platform (MSD, Rockville, Maryland) in technical duplicates or triplicates. Standard curves were created using the MSD Mesoscale Discovery Workbench Toolbox to benchmark Aβ concentrations in the samples. A 4-parameter logistic curve was used to fit standards and calculate the concentration for unknowns and Aβ controls. Aβ standards for the calibration curve were measured in duplicate and were set in serial in 1:4 dilutions. The upper and lower limits of detection were set as 2.5 standard deviations from the bottom and top calibrator. The calculated Aβ amounts were then normalized to the total extracted protein levels from each sample were determined using the Bradford assay.

#### P3 (Beta-Amyloid 17-42 peptide) preparation and injection

HFIP-treated Beta-Amyloid 17-42 peptide (P3) (Cambridge Biosciences) was dissolved in DMSO and vortexed briefly to yield a 100μM solution. The stock solution was aliquoted as 5μl in individual tubes and kept at −80°C. Just before the injections, stock solutions were 1:10 serially diluted in Phosphate buffered saline (PBS) to obtain a 10 nM solution. The injections were carried out with a Pneumatic PicoPump (WPI) and glass capillary needles (Science Products Gmbh) prepared with a Micropipette Puller (Shutter Instruments). Larvae (5 dpf) were anesthetized using 4% Tricaine (42 mg/L, Sigma) 30 minutes before injections. Larvae were immobilized dorsal up in 2% low melting point agarose (Thermo Fischer) in fish water on a small petri dish lid. 1 nl of the 10 nM Beta-Amyloid 17-42 peptide solution was injected into the hindbrain ventricle. For controls, 1nL of 1x PBS was injected instead. Success of all injections was confirmed by judging the inflation of the ventricles. Larvae were removed from the agar, put into fresh water for 20 minutes to recover from the Tricaine, and transferred into a 96 square-well plate to undergo sleep/wake behaviour tracking (see method details: behavioural experiments**)**.

### Quantification and statistical analysis

Effect sizes were calculated using the dabest estimation package implemented in MATLAB^89^ by creating multiple bootstrap resamples from the dataset, computing the effect sizes on each of these resamples, and determining the 95% CI from these bootstrapped resamples.^89^ All other statistical tests were done using MATLAB (The Mathworks, R2019a). Data was tested first for normality using the Kolmolgorov-Smirnov Normality test, outliers were detected removed using Grubb’s test at p<0.01, and the data was analysed with one-way ANOVA or Kruskal-Wallis followed by Tukey’s or Dunnett’s post-hoc test. A two-way ANOVA was used to calculate the interaction statistics for the drug x genotype experiments.
