# Supplementary material for: Genetic and chemical disruption of amyloid precursor protein processing impairs zebrafish sleep maintenance
Source: iScience. 2024 Jan 11;27(2):108870. doi: 10.1016/j.isci.2024.108870 (PMC10839650; doi:10.1016/j.isci.2024.108870)
Supplement: Document S1. Figures S1–S6 [file mmc1.pdf]

iScience, Volume *27*

Supplemental information

Genetic and chemical disruption of amyloid precursor protein processing impairs zebrafish sleep maintenance

Güliz Gürel Özcan, Sumi Lim, Thomas Canning, Lavitasha Tirathdas, Joshua Donnelly, Tanushree Kundu, and Jason Rihel

Figure S1

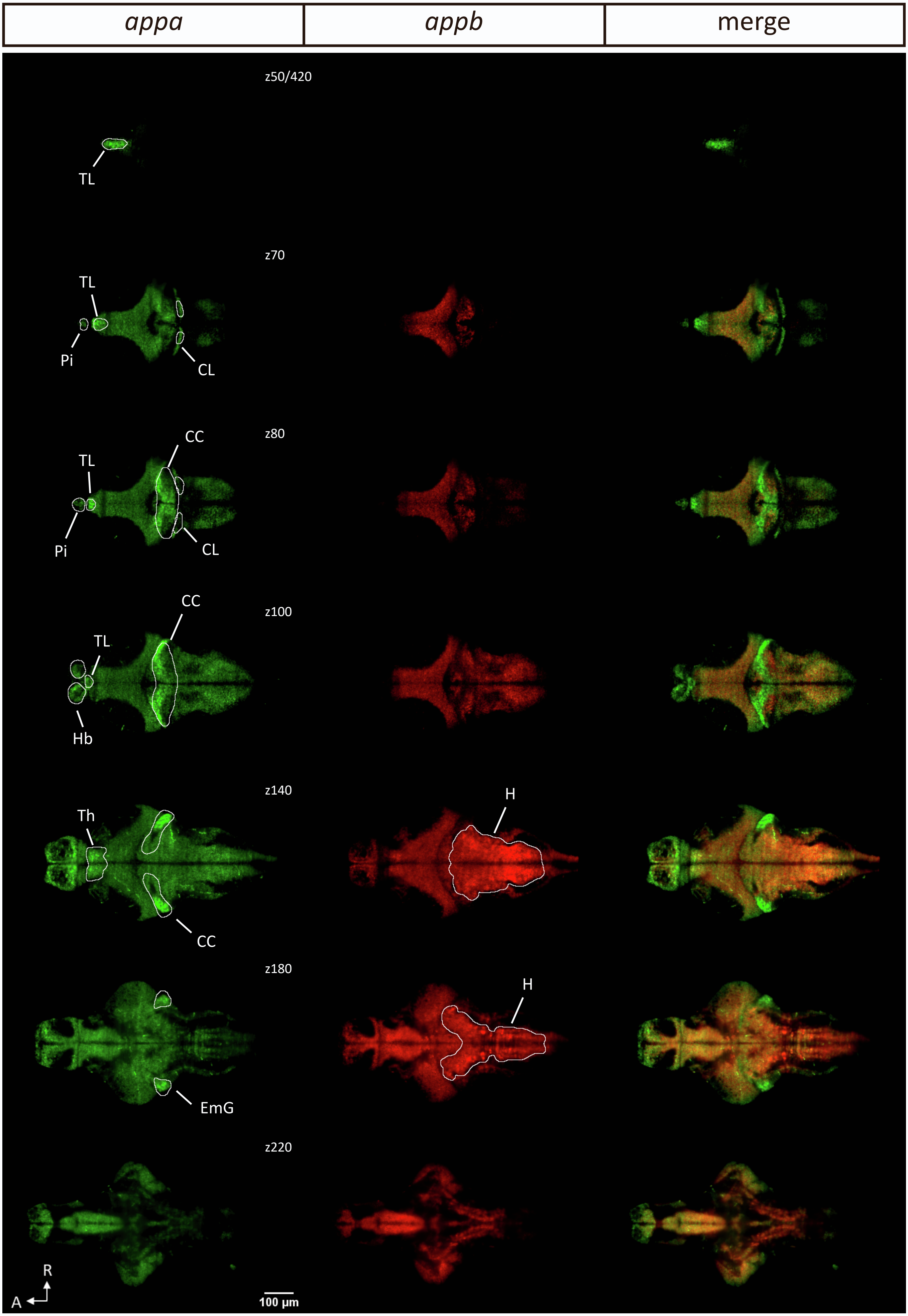
appa
appb
merge
z50/420
TL
z70
TL
Pi
CL
z80
CC
TL
Pi
CL
z100
CC
TL
Hb
z140
Th
CC
H
z180
H
EmG
z220
R
A
100 µm

Supplemental Figure S1: *appa* and *appb* have differential brain expression, related to Figure 1.

As detected by multiplexed hybridization chain reaction (HCR), *appa* (green) and *appb* (red) are expressed differentially at 5 dpf. Dorsal view of several individual z-planes (z50, 70, 80, 100, 140, 180, 220) from a single representative brain (n=22 larvae) are shown. Brains were registered to the ZBB brain atlas using the *gad1b*-HCR reference brain, and outlines of anatomical regions are based on the Z-brain binary masks (Randlett et al., 2015). For the habenula, torus longitudinalis, and pineal gland, the outlines were translated to account for suboptimal registration. The hindbrain outline was generated manually. TL = Torus longitudinalis, Pi = Pineal, CL = Cerebellum – caudal lobe, CC = Corpus cerebelli, Hb = Habenula, EmG = Eminentia granularis, H = Hindbrain; A = Anterior, R = right.

Supplemental Figure S2

A)

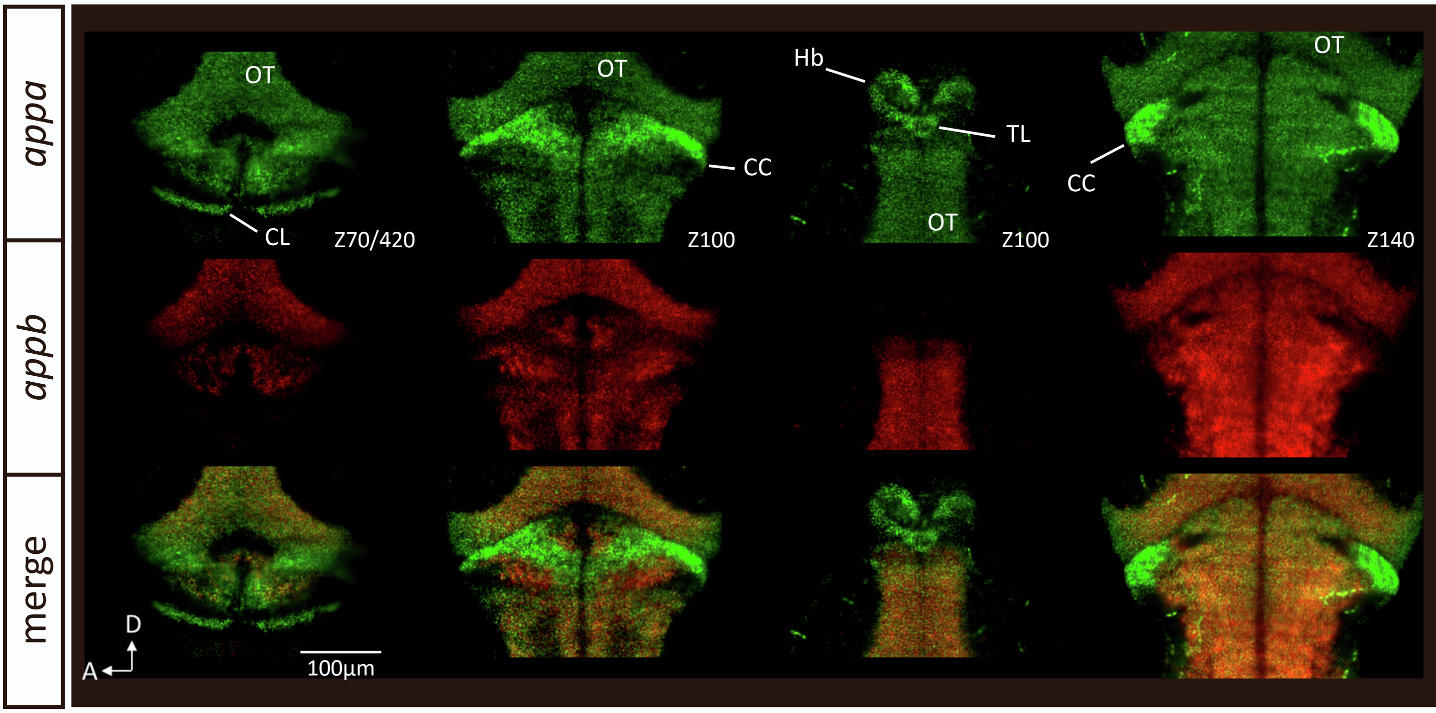

B)

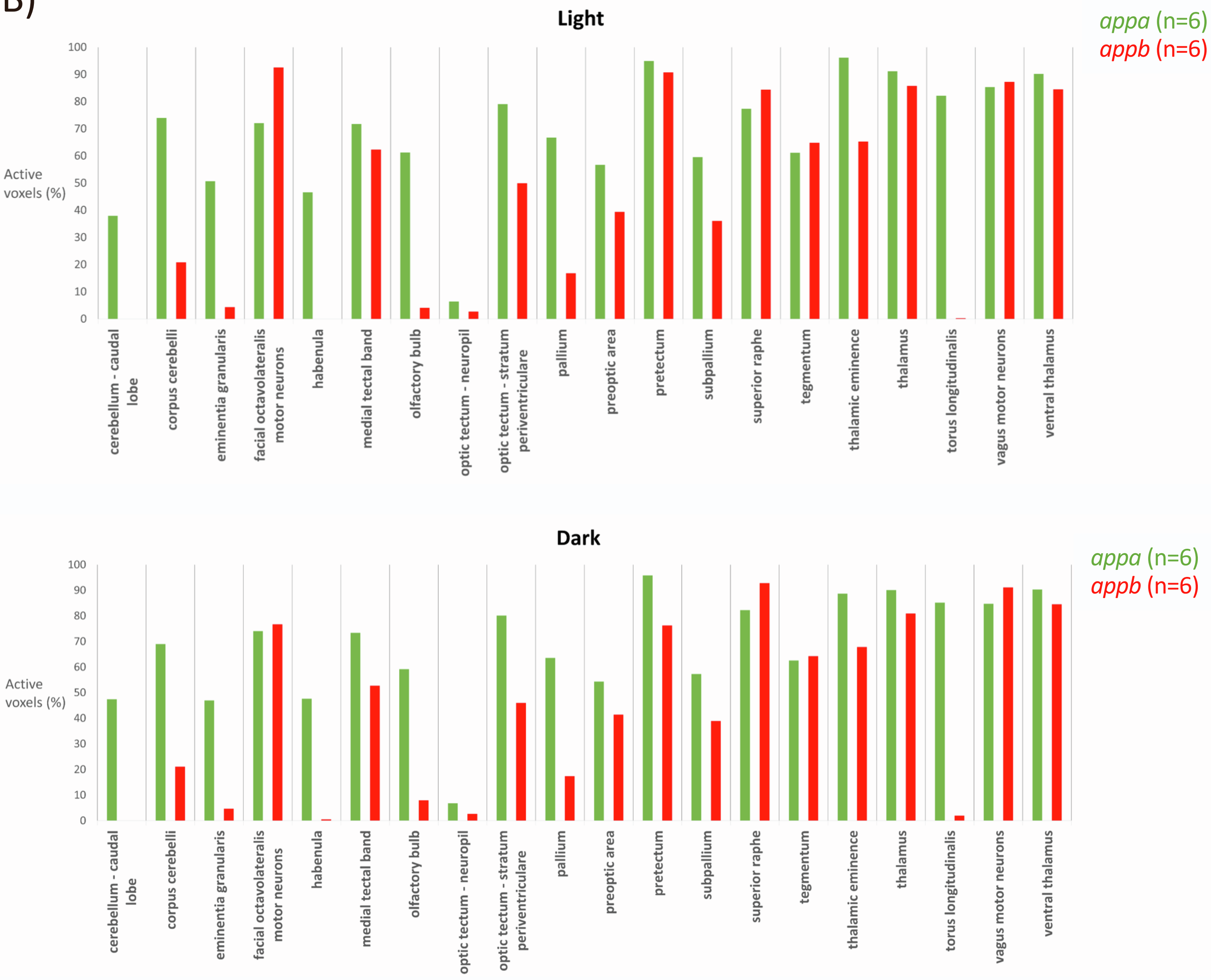

Supplemental Figure S2: *appa* and *appb* brain expression, related to Figure 1.

A) Cropped and expanded dorsal views showing *appa* (green) and *appb* (red) expression visualised with multiplexed HCR. Several individual z-planes (z70, 100, 140) from a single representative brain (n=22) are shown. Some weak expression of *appb* in the habenula is not visible due to thresholding. OT= Optic Tectum, TL = Torus longitudinalis, CL = Cerebellum – caudal lobe, CC = Corpus cerebelli, Hb = Habenula; A = anterior, D = Dorsal.

B) Mean % active voxel values of multiplexed in situ hybridisation signals for *appa* (green) and *appb* (red) presented from light (top panel, n=6) & dark (bottom panel, n=6) conditions across brain regions.

Supplemental Figure S3

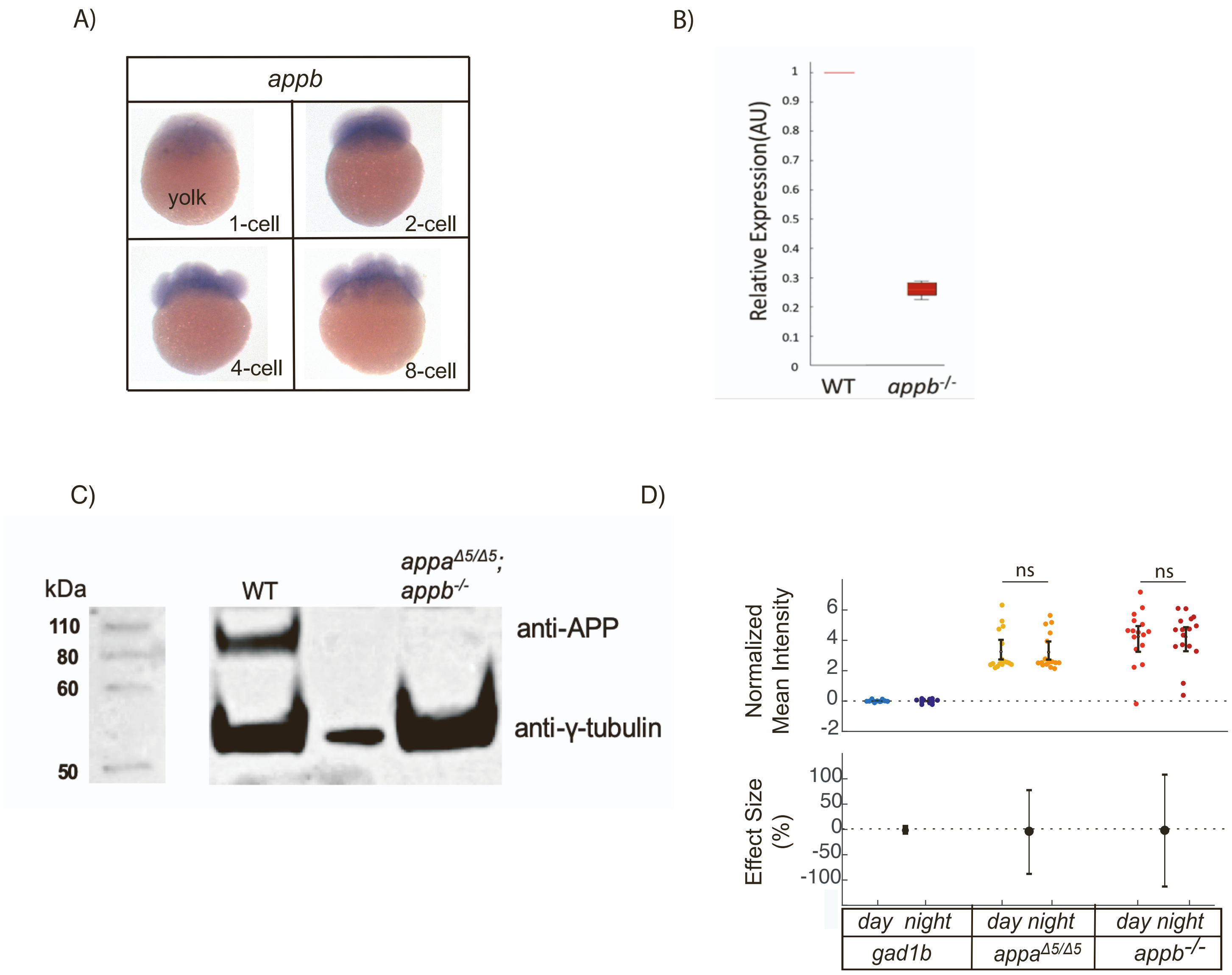

Supplemental Figure S3: *appa* and *appb* expression, related to Figure 1.

A) As detected by in situ hybridization (ISH), *appb* is expressed in early development (1-8 cell stage, dark purple staining), indicating that it is maternally deposited. **B)** qRT-PCR of *appb* transcript levels from 5dpf WT and *appb*$^{-/-}$ mutant larvae, indicating non-sense mediated decay. **C)** The uncropped Western blot analysis from Figure 1E of APP in brain homogenates from wildtype (WT) and *appa*$^{\Delta 5/\Delta 5}$*; appb*$^{-/-}$ double mutants. An empty column was left in between samples to avoid spill-over, but was cropped in Figure 1E for clarity. **D)** Mean intensity of *appa* and *appb* expression visualized with multiplexed in situ hybridisation, normalized to the reference channel *gad1b* at 5 dpf during the day and night. Each dot represents one brain image. N=16 per condition.

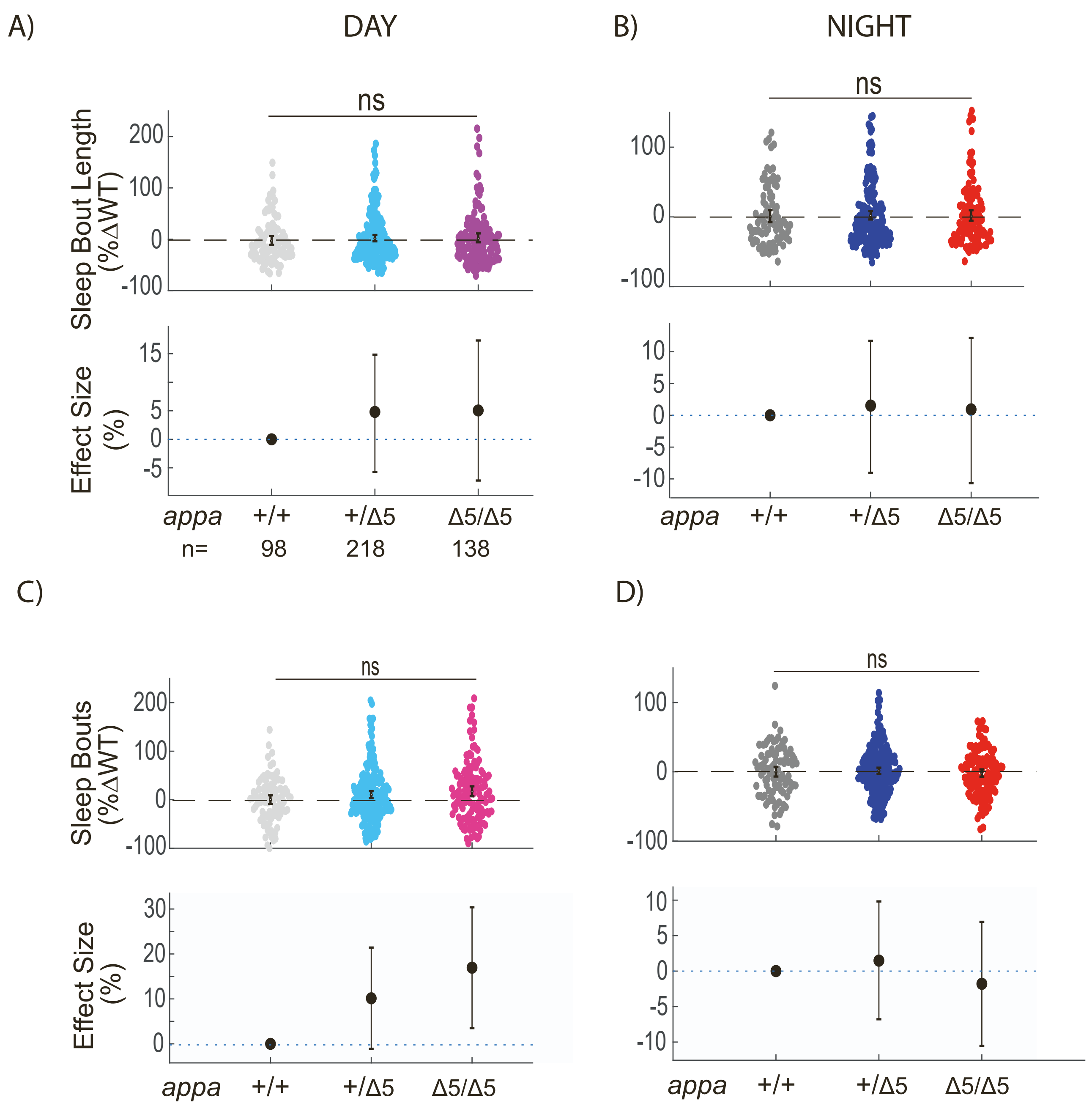
A)
DAY
B)
NIGHT
ns
Sleep Bout Length (%ΔWT)
200
100
0
-100
Effect Size (%)
15
5
0
-5
10
-10
appa
+/+
+/Δ5
Δ5/Δ5
n=
98
218
138
C)
D)
Sleep Bouts (%ΔWT)
30
20

Figure S4

Supplemental Figure S4. Sleep parameters of *appa* mutants, related to Figure 2.

A) Day sleep length, **B)** Night sleep length, **C)** day sleep bout number and **D)** night sleep bout number of *appb*$^{-/-}$ mutants normalized to WT siblings across N=5 independent experiments. At top, each animal (dots) is normalized to the mean of their experimentally-matched WT data. At bottom, the effect size ± 95%CI relative to the WT mean are shown. $^{ns}p>0.05$, Kruskal-Wallis followed by Tukey's test. n = the number of larvae.

Supplemental Figure S5

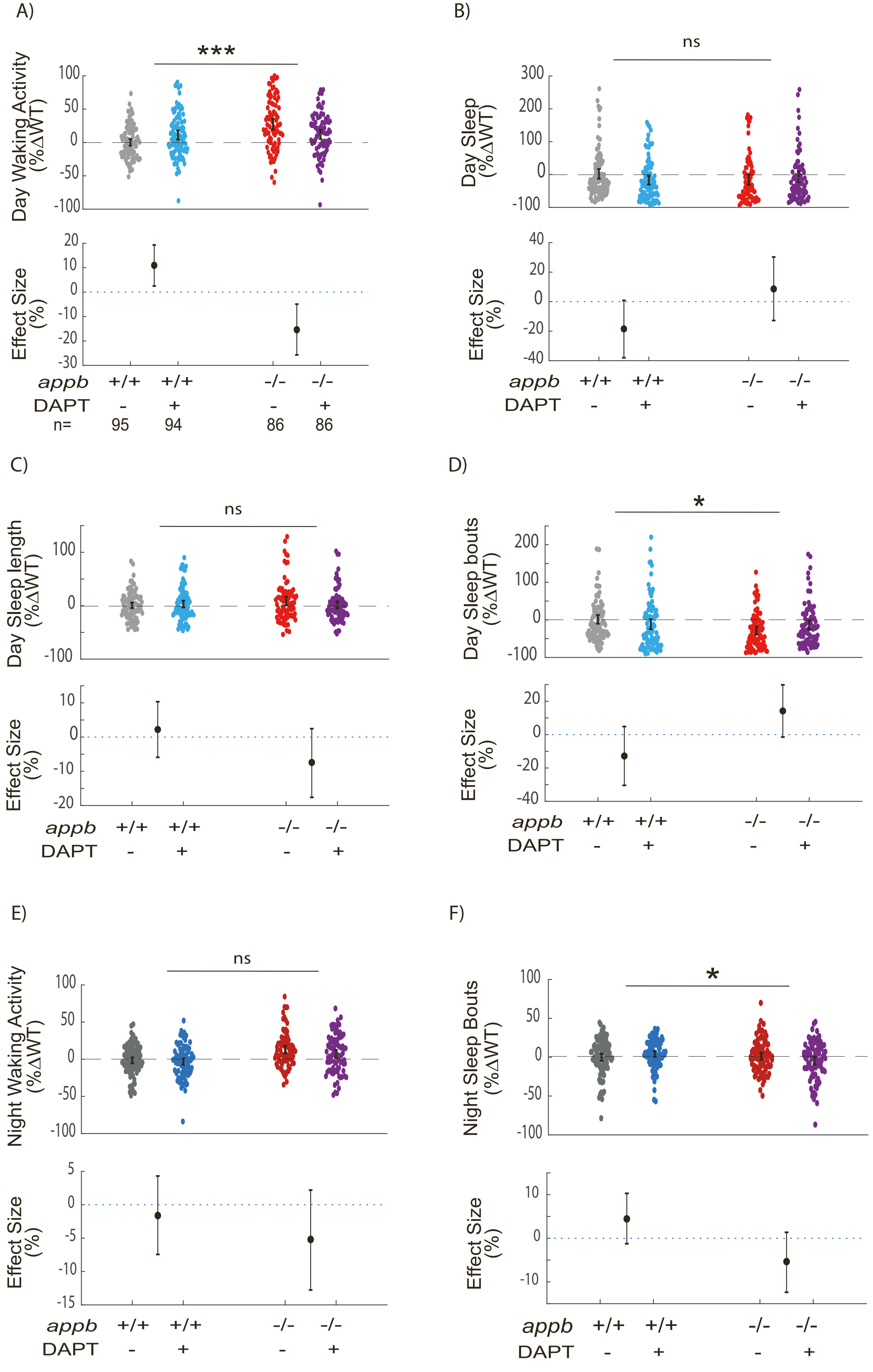

Supplemental Figure S5. The y-secretase inhibitor DAPT affects multiple behaviors in WT and *appb*$^{-/-}$ mutants, related to Figure 4.

A) Average day waking activity, **B)** day sleep, **C)** day sleep bout length, **D)** day sleep bout number, **E)** night waking activity, and **F)** night sleep bout number of WT and *appb*$^{-/-}$ mutants exposed to either DAPT or DMSO vehicle. At top, each dot represents a single larva normalized to the mean of the experimentally-matched WT fish in DMSO, and error bars indicate ± SEM. At bottom, the effect size and 95%CI are plotted. n = the number of larvae. Data is pooled from N =4 independent experiments. $p>0.05$, *$p\leq0.05$, **$p\leq0.01$, 2-way ANOVA. p values indicate drug x genotype interaction.

Supplemental Figure S6

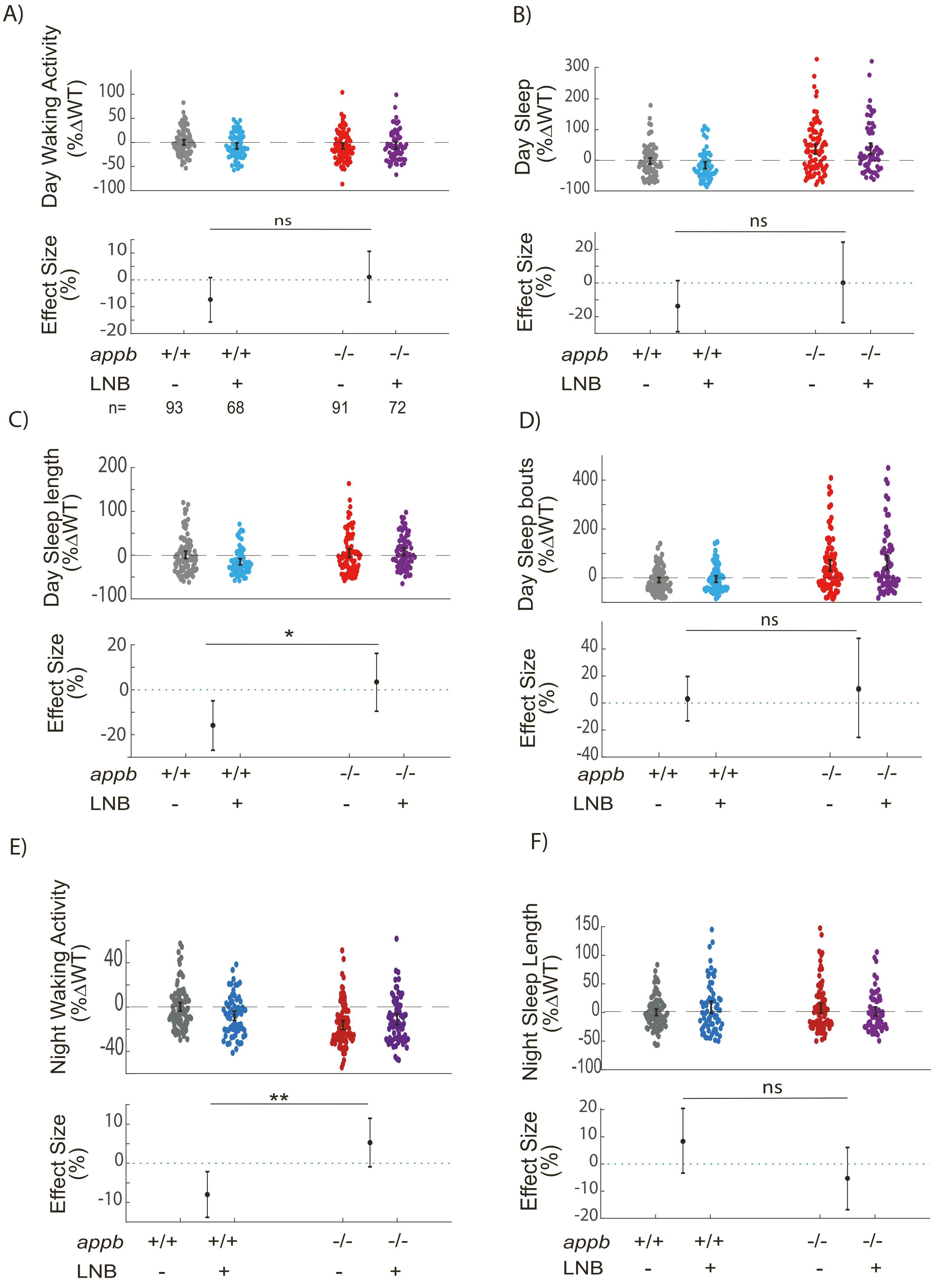

Supplemental Figure S6. The β-secretase inhibitor Lanabecestat alters sleep at night in WT but not in *appb*$^{-/-}$ mutants, related to Figure 5.

A) Average day waking activity, **B)** day sleep, **C)** day sleep bout length, **D)** day sleep bout number, **E)** night waking activity, **F)** night sleep bout number of WT and *appb*$^{-/-}$ mutants exposed to 0.3 μM Lanabecestat or DMSO vehicle. At top, each dot represents a single larva normalized to the mean of the experimentally-matched WT fish in DMSO, and error bars indicate ± SEM. At bottom, the effect size and 95%CI are plotted. n = the number of larvae. Data is pooled from N =4 independent experiments. p>0.05, *p≤0.05, **p≤0.01, 2-way ANOVA. p values indicate drug x genotype interaction.
